# A structural foundation for studying chlamydial polymorphic membrane proteins

**DOI:** 10.1128/spectrum.03242-23

**Published:** 2023-10-26

**Authors:** Abigail M. Debrine, P. Andrew Karplus, Daniel D. Rockey

**Affiliations:** 1 Department of Biomedical Sciences, Oregon State University, Corvallis, Oregon, USA; 2 Department of Biochemistry and Biophysics, Oregon State University, Corvallis, Oregon, USA; LSU Health New Orleans, New Orleans, Louisiana, USA

**Keywords:** *Chlamydia*, polymorphic membrane protein, AlphaFold, autotransporter, sexually transmitted infection, trachoma

## Abstract

Polymorphic membrane proteins (Pmps) are chlamydial cell surface proteins with signature repeating FxxN and GGA (I, V, L) tetrapeptide motifs. These autotransporter proteins are key immune targets and mediators of host-microbe interactions but by unknown mechanisms. AlphaFold predictions reveal that Pmp passenger domains have two distinct β-helical parts: a C-terminal globular “Pmp middle region” that forms six β-helical rungs, and an N-terminal highly-regular “Pmp repeat region” that is from 9 to 25 rungs long. In the repeat region, paired FxxN and GGA (I, V, L) motifs – or close variants – are fully buried and packed together to form novel β-helical rungs from which variable-length loops extend out from one face. Similar motifs in proteins from diverse organisms are predicted to form analogous structures. In Pmps, the structures imply that the tetrapeptide motifs are not directly involved in host-cell adhesion, but position protein loops on a common face where they can interact with ligands important for infection. These structural descriptions provide a guide for mutagenesis studies to identify those parts of Pmps involved in infection.

**IMPORTANCE:**

Infections by bacteria in the genus *Chlamydia* cause a range of widespread and potentially debilitating conditions in humans and other animals. We analyzed predicted structures of a family of proteins that are potential vaccine targets found in all *Chlamydia* spp. Our findings deepen the understanding of protein structure, provide a descriptive framework for discussion of the protein structure, and outline regions of the proteins that may be key targets in host-microbe interactions and anti-chlamydial immunity.

## INTRODUCTION

Species within the genus *Chlamydia* are obligate intracellular gram-negative bacteria that cause disease in a wide variety of animal species. *Chlamydia trachomatis* infection of the human genital tract is the most common reportable disease in the USA and the most common bacterial sexually transmitted infection (STI) worldwide (1). Infections are often asymptomatic and undiagnosed (2), but persistent or repeated infections can lead to serious sequelae, including pelvic inflammatory disease, infertility, and potentially fatal ectopic pregnancies. *C. trachomatis* can also infect the eye, causing the disease trachoma, which is the leading cause of infectious blindness worldwide (1, 3, 4). Some chlamydial pathogens of veterinary significance can cause serious zoonotic diseases in humans (5). Antibiotic therapy remains the primary tool for the control of human chlamydial disease, as there are no available vaccines against any human chlamydial infection (1).

Although the diseases caused by and hosts infected by *Chlamydia* spp. are diverse, much of the basic chlamydial infection process is highly conserved (6). There is significant conservation of the constituency of the chlamydial outer membrane complex, which is a primary point of contact between the microbe and its host cell environment. One group of potential virulence factors common to all chlamydial species is the polymorphic membrane proteins (Pmps), a large family of surface-exposed outer membrane proteins (7). The function of Pmps is unclear, but some members have been implicated in attachment, invasion, and anti-chlamydial host immune responses (8 – 11). Expansion of the Pmp family is an indicator of unique importance, as genome reduction is evident for almost every other class of chlamydial proteins.

**Fig 1:**
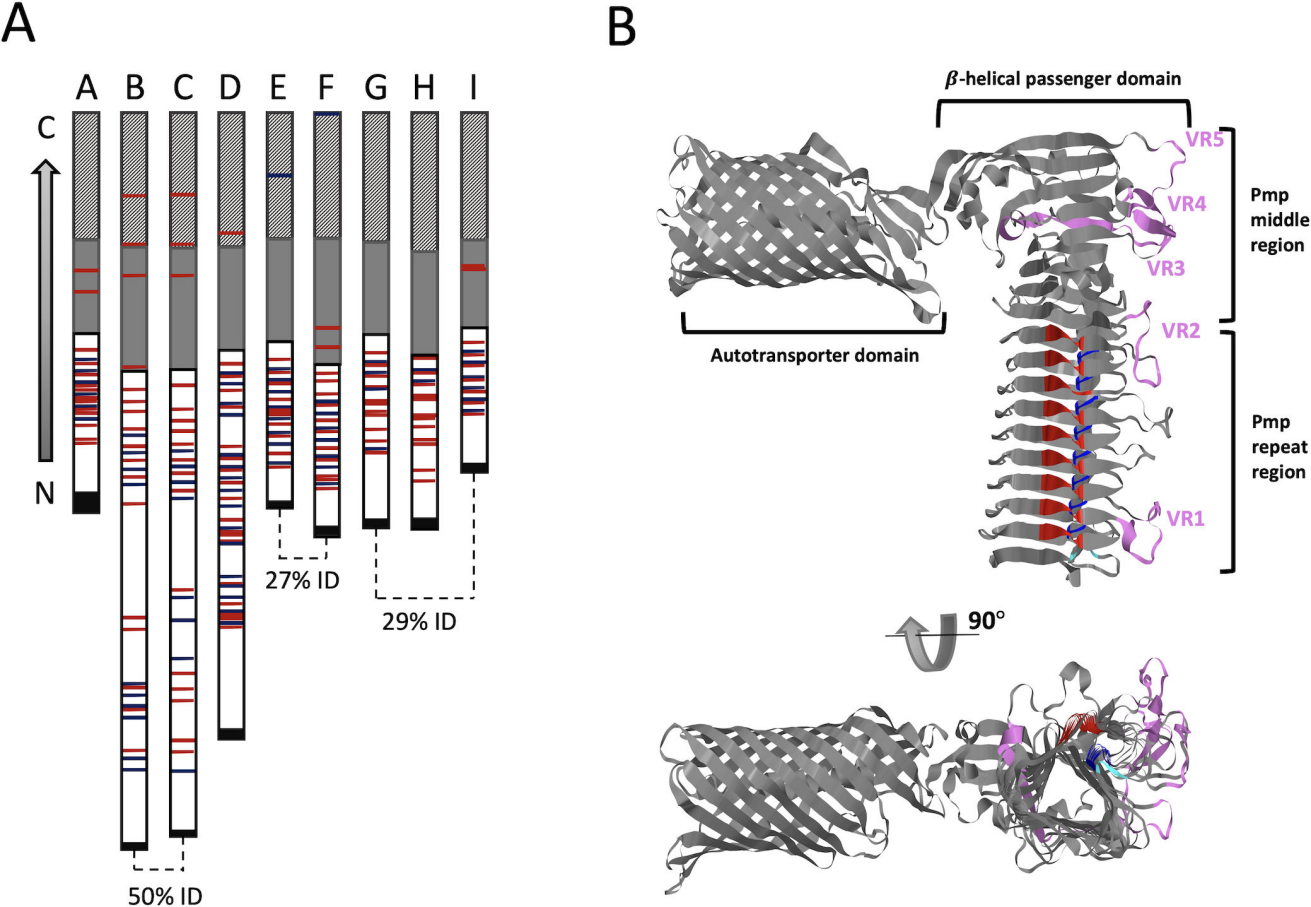
Overall structure of chlamydial Pmps. (**A**) Domain organization of the nine Pmps (A through I) from *C*. t*rachomatis*, with percent sequence identity noted for more closely related protein pairs. Shown to scale in each protein are regions defined in the PFAM database [https://www.ebi.ac.uk/interpro/entry/pfam/PF02415/ (18)]: the autotransporter region (striped; PF03797), the middle region (gray), and the repeat-containing region (open). The predicted signal peptide is indicated in black. The tetrapeptide motifs FxxN (red) and GGA (I, V, L) (blue) motifs are indicated in each protein. (**B and C**) Orthogonal views of a ribbon representation of the Alphafold2 predicted structure of *C. trachomatis* PmpE D/UW/3 (C*t*PmpE). Regions corresponding to the three PFAM-identified domains are indicated. The N-terminal passenger domain encompasses both the middle region and the repeat-containing region. The FxxN motifs (red), GGA (I, V, L) motifs (blue), and the five tropism-predicting variable regions [VR1-VR5; indigo (17)] are also highlighted.

The Pmps are a distinct subfamily of the large single-chain autotransporter proteins of the type V secretion system of Gram-negative bacteria (12, 13). Autotransporter proteins are all composed of (i) a signal peptide that directs the protein to the periplasm, (ii) an approximately 300 amino acid C-terminal β-barrel autotransporter domain that is embedded in the outer membrane, and (iii) an N-terminal passenger domain that, in most cases, adopts a β-helix fold and that is extruded through the β-barrel out into the extracellular space in a process modeled to be driven by its directional folding starting from the C-terminal end (13) (Fig. 1). Based on sequence comparisons, the passenger domain of Pmps can be further divided into a C-terminal ~200 residue “Pmp middle region” and a variable-length N-terminal “Pmp repeat region” characterized by repeats of the tetrapeptide motifs FxxN and GGA (I, V, L) that are often paired (9, 14). For the nine *C. trachomatis* Pmps (*Ct*Pmps)*,* this portion ranges from about 400 to 1,200 residues long (Fig. 1A). Mutational studies have shown that the repeat motifs may be necessary for adhesion (15), but it is not known if their role is direct or indirect (16). Our laboratory has recently identified variations in the *C. trachomatis* PmpE sequence that correlate with tissue tropism by serovars within the species (17).

The artificial-intelligence-based program AlphaFold, and its improved version, Alphafold2, have revolutionized modern protein structure prediction; from sequence alone, the correct fold and coordinates of a protein are generally predictable within ~2 Å of the true structure (19, 20). As no experimental structures are available for any Pmp, we used the AlphaFold2 predicted structure of PmpE (Fig. 1B) to gain insight into the roles of variable regions that correlate with tissue tropism. The predicted structure includes the expected β-barrel autotransporter domain, as is common to type V secreted proteins (12, 13), and an N-terminal passenger domain in the form of a lengthy β-helix that is exported to the bacterial cell surface [ref (16), Fig. 1B]. Using the predicted structure, we found that the PmpE variable regions correlating with tissue tropism reside in surface-exposed loops (Fig. 1B), and we hypothesized that the loops bind differentially to tissue-specific host ligands, affecting host tropism by different strains (17).

In this report, we provide a foundation for studies of the Pmp proteins by describing key features of the AlphaFold2-predicted chlamydial Pmp structures. The structures, each predicted with high confidence by the program, show that the characteristic tetrapeptide motifs FxxN and GGA (I, V, L) are core elements of a regular β-helical framework within each Pmp passenger domain. We also identify noncanonical motifs, similar in sequence to FxxN and GGA (I, V, L), that perform the same role. While these tetrapeptide motifs are fully buried, they provide a consistent platform from which highly exposed loops extend out from the β-helical passenger domain such as would allow them to be involved in adhesion. This predicted structural information provides a powerful basis for guiding mutation-based studies of Pmp function and immunogenicity.

## RESULTS

### AlphaFold2 structure predictions are robust and relevant for the entire *Chlamydia* spp. Pmp family

**Fig 2:**
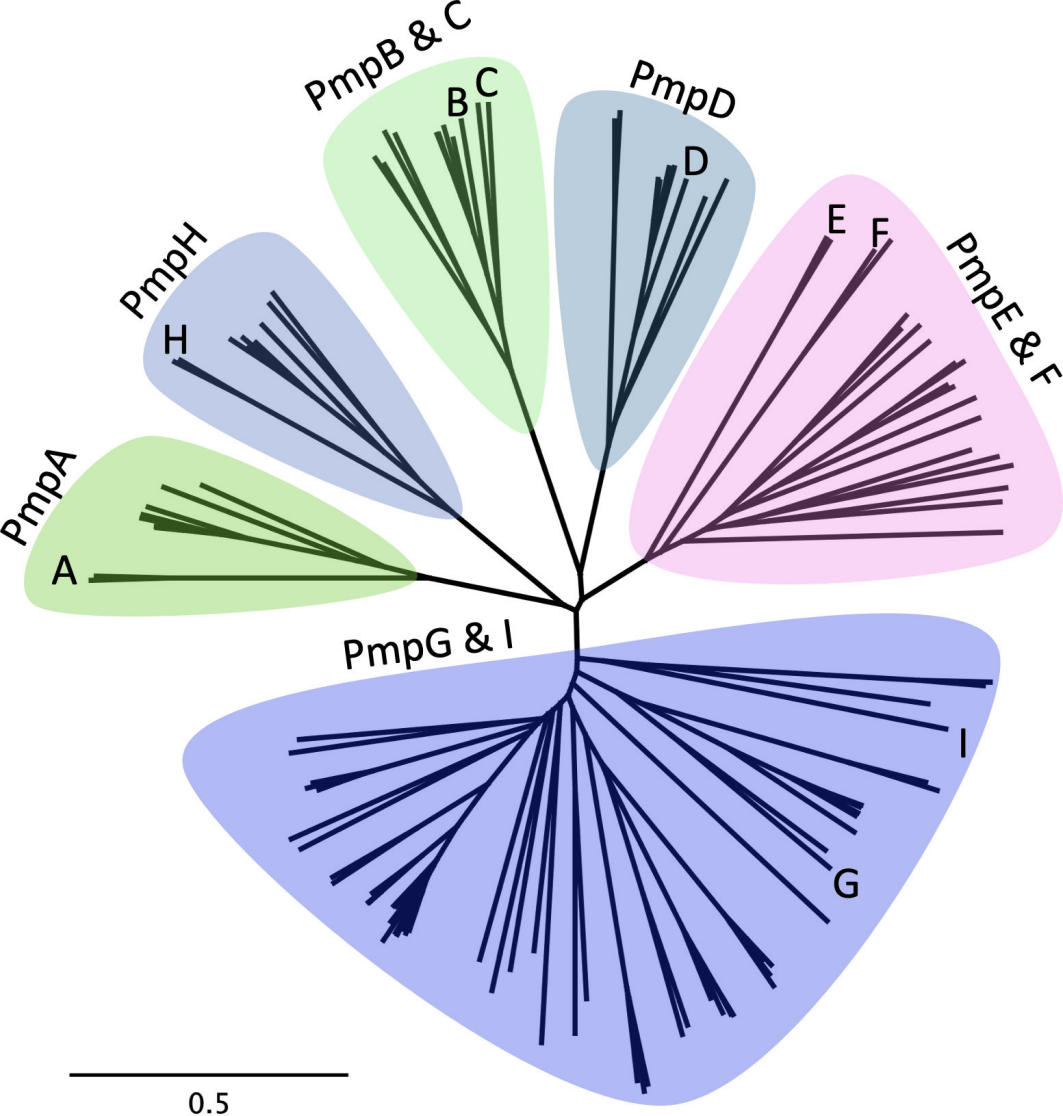
Relatedness tree of chlamydial Pmp sequences. An unrooted phylogenetic tree is shown that includes Pmp sequences from the 10 chlamydial species identified by Vasilevsky et al. (16), except those that appeared to be incomplete (either having less than 500 residues or not having all three regions). Each clade is named based on the *C. trachomatis* Pmp or Pmps contained in the clade. The scale bar represents the estimated number of substitutions per site. A rooted tree showing all Pmps included in this analysis is presented in Fig. S2.

We explored the overall relatedness of the larger family of chlamydial Pmps by first assembling a phylogenetic tree of all Pmps from 10 species, as organized by Vasilevsky et al. (16). All examined Pmps cluster into six large clades, each of which contains one or two members of the *Ct*Pmp family (Fig. 2). These analyses are very similar when conducted on whole Pmps (Fig. 2) or just the passenger domains (not shown). Given that the nine *Ct*Pmps span the diversity of *Chlamydia* spp. Pmps, we accessed the AlphaFold2 predicted structures for all nine *Ct*Pmps. These structures each shared the overall structural organization identified for PmpE, with a C-terminal β-barrel autotransporter domain connected by a flexible linker to a β-helical passenger domain (Fig. 1B; Fig. S1). The differences in the length of the β-helical passenger domain evident in the different Pmps are largely due to the varying length of the repeat region (Fig. 1A). Additionally, the characteristic tetrapeptide motifs are found in consistent positions within rungs of the repeat region β-helix of every *Ct*Pmp (Fig. 1B; Fig. S1).

**Fig 3:**
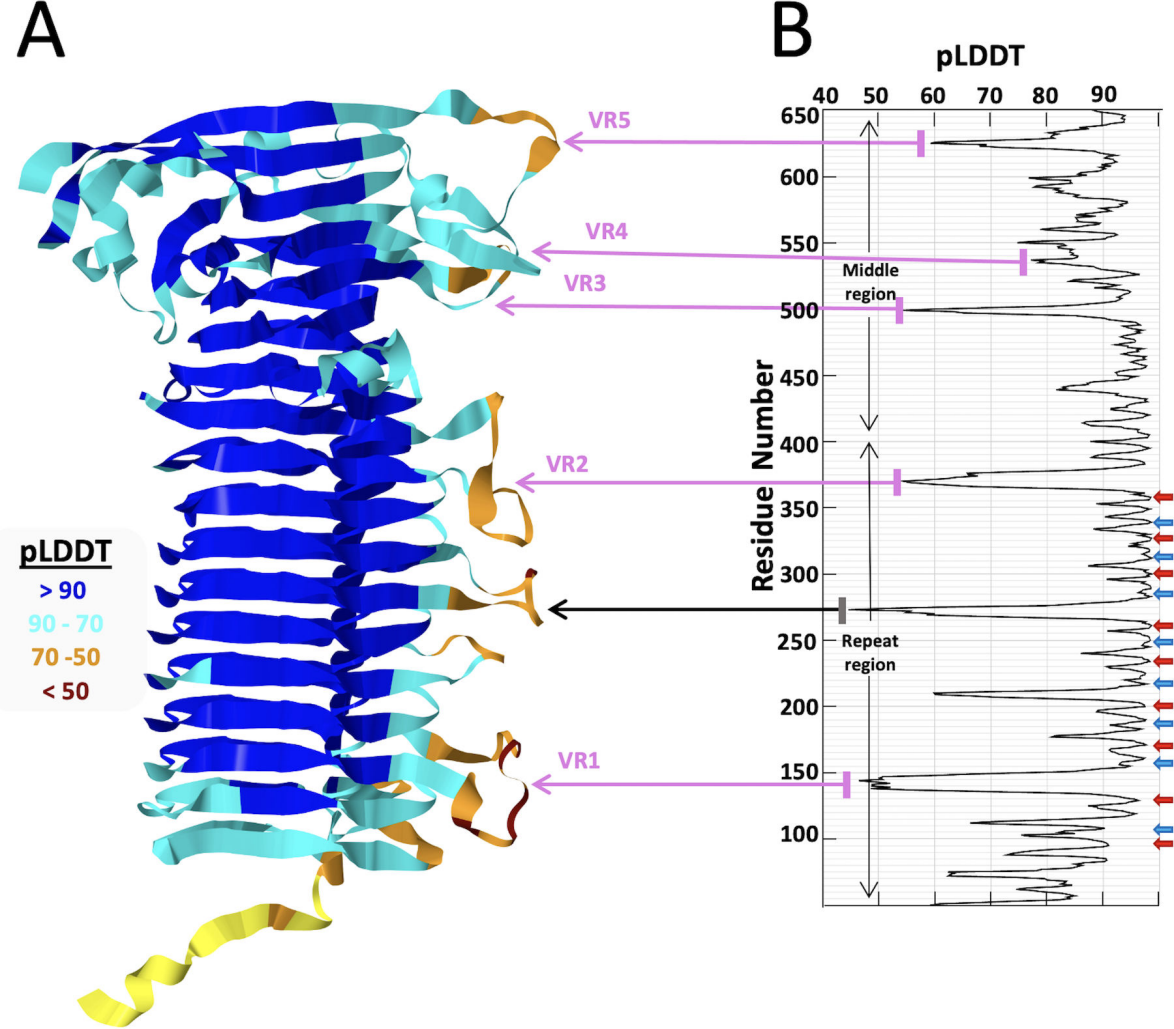
pLDDT confidence values for the predicted passenger domain structure. (**A**) Alphafold2 predicted model of the passenger domain of PmpE with color coding that reflects the pLDDT values (color key in figure). (**B**) The pLDDT plot for the passenger domain of PmpE, aligned with the model in panel A. FxxN and GGA (I, V, L) motif locations are indicated with red and blue arrows, respectively. Also highlighted are the VR1–VR5 variable regions that correlate with tissue tropism (pink arrows), and one additional region that has pLDDT ≤50 (gray arrow).

AlphaFold2 reports a “predicted local distance difference test” (pLDDT) score that provides a measure of the confidence of the predicted structure on a residue-by-residue basis, with pLDDT scores of >90 indicating an expected high accuracy model (20). The pLDDT values across the predicted passenger domain structure for PmpE (Fig. 3) demonstrate that the core parts of the β-helix are confidently predicted, with the FxxN and GGA (I, V,L) motifs located in regions having the highest pLDDT values. In contrast, loops extending out from the core β-helical structure have markedly lower pLDDT values, indicating a generalized lack of definable structure in these regions (Fig. 3). Similar pLDDT profiles are present in each of the predicted *Ct*Pmp structures (data not shown).

### Features of the predicted Pmp passenger domain fold

**Fig 4:**
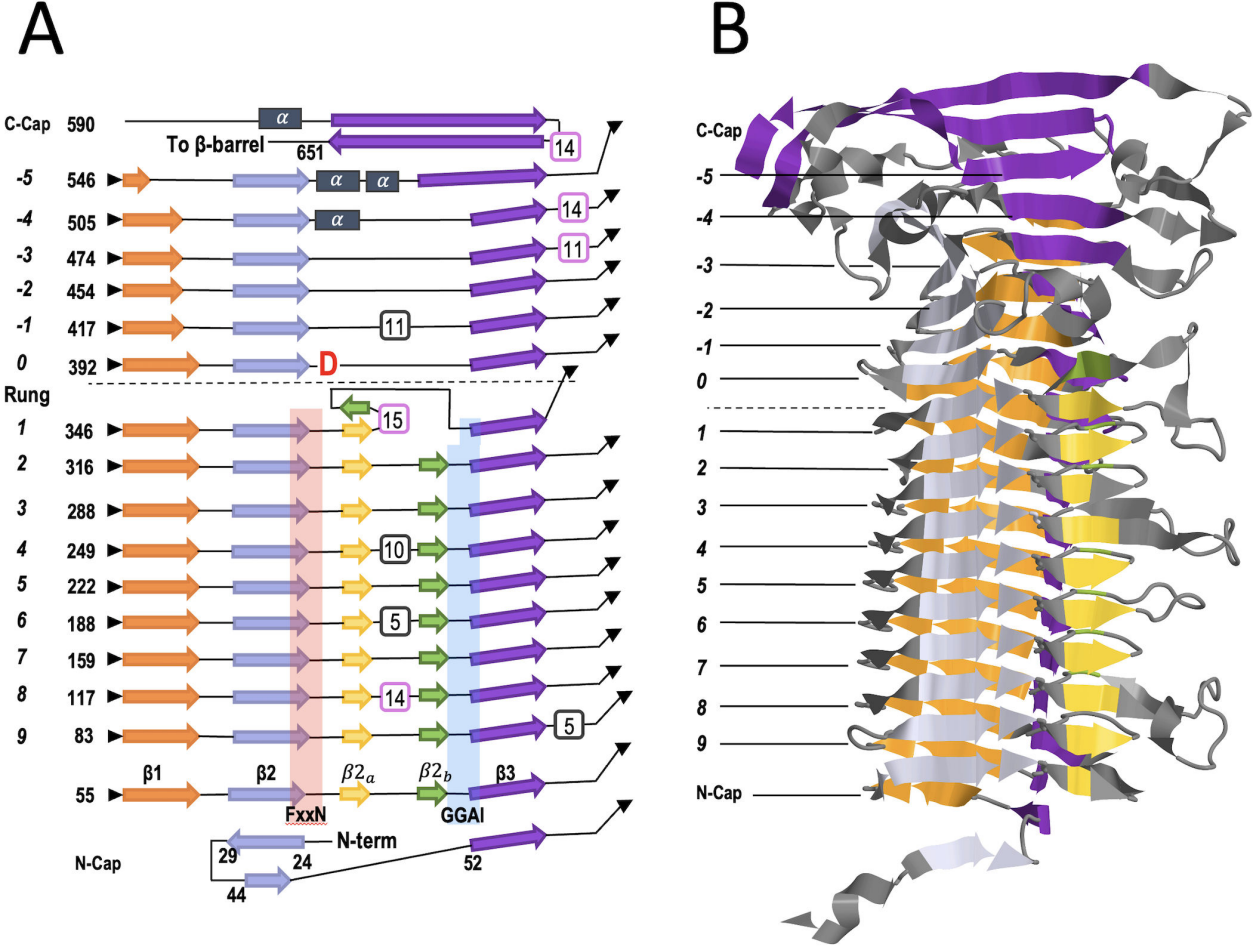
Overall topology of the predicted passenger domain structure. (**A**) Topology diagram of the β-helical passenger domain of PmpE, with the N-terminus at the bottom and C-terminus at the top. Residue positions are indicated for the start of each β-helical rung and the end of the passenger domain. β-strands (arrows) and α-helices (grey rectangles) are shown with unique colors for each β-sheet. Pastel shading indicates the locations of the FxxN (red) and GGA (I, V, L) (blue) motifs. The boxed numbers indicate loops longer than five residues that extend from the β-helical core, with those corresponding to VR1–VR5 regions (17) boxed in pink. The bold red D represents a fully conserved Asp residue that defines *Rung 0*, the transition between the middle region and the repeat region. These regions are separated by a horizontal dotted line. Rungs above *Rung 0*, moving toward the C-terminus are indicated with negative numbers, while rungs below *Rung 0*, moving toward the N-terminus, are indicated with positive numbers. (**B**) Ribbon diagram of the Alphafold2 predicted model of the *Ct*PmpE (407–657) passenger domain, matching the scale and β-sheet coloring of panel A and with Rungs (italics) and the N- and C-cap elements labeled.

The overall predicted structure common to the Pmp passenger domains is a long right-handed β-helix that includes two distinct parts: the Pmp middle region forms a partly globular and irregular β-helix and the more N-terminal Pmp repeat region forms a highly regular β-helix (Fig. 1B; Fig. S1). Since autotransporter passenger domains fold from the C- to the N-terminal end (13), we describe the structure in that order. A topology diagram of the whole passenger domain of PmpE (Fig. 4; Fig. S3) provides an overview of the fold organization and includes a proposed common numbering system for describing the β-helical rungs of Pmp passenger domain structures. A transition rung between the more globular Pmp middle region, and the very regular Pmp N-terminal repeat region is defined as *Rung 0*. This rung is uniquely identifiable as it contains a central Asp residue (red D in Fig. 4) that is found in every one of the over 120 Pmps representing all Pmps from 10 *Chlamydia* spp. (Fig. S2 and S4D). Negative rung numbers (from *Rung −5* through *Rung −1*) are given to rungs on the C-terminal side that make up the Pmp middle region, and positive rung numbers (starting with *Rung 1*) are given to subsequent rungs in the N-terminal direction that make up the Pmp repeat region. The next paragraphs use this nomenclature in describing features of the Pmp middle and repeat regions.

The Pmp middle region is attached by a short flexible linker to the transmembrane β-barrel, and the region starts with two long antiparallel β-strands that form a wide structural cap (C-cap in Fig. 4). Then the first two rungs of the Pmp middle region (*Rung −5* and *Rung −4*; Fig. 4) are irregular β-helical layers, and these are followed by three regular β-helical rungs (*Rungs -3, –2,* and *−1*; Fig. 4) with a narrower triangular cross section. The final helical rung of the Pmp middle region is the foundation on which the repeat region folds and includes the highly conserved Asp residue mentioned above. (Fig. 4; Fig. S3). These elements, including the exact number of rungs, make up the Pmp middle region topology that is present in the predicted structures of all nine *Ct*Pmps.

**Fig 5:**
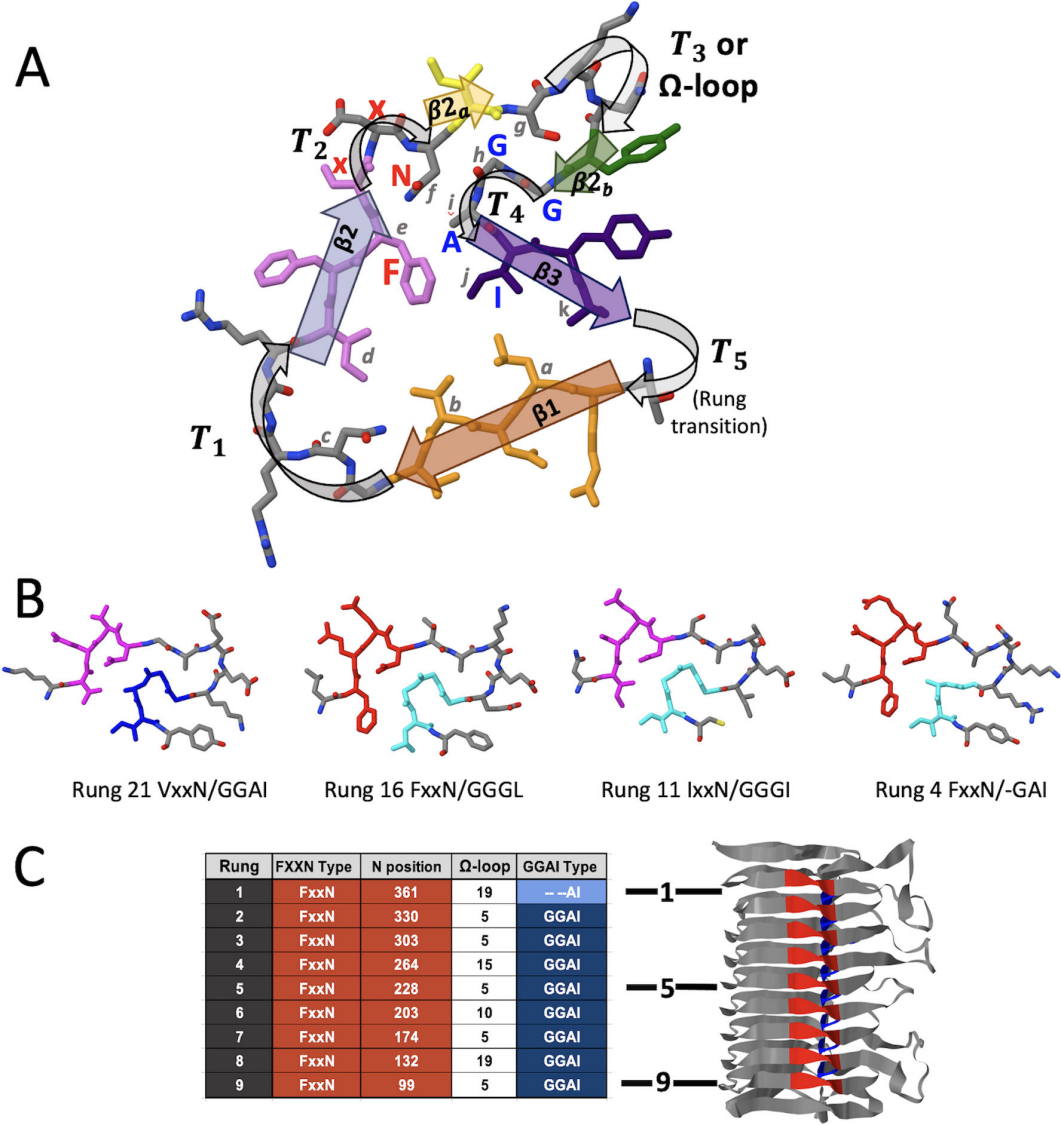
Common structure of rungs within the β-helical repeat region. (**A**) Stick model of a representative rung (*Rung 2* of *Ct*PmpE; residues 287–314) highlighting common structural elements. The β-strands β1 (orange), β2 (blue), and β3 (violet) form the main triangular cross-section short β2_a_ (yellow) and β2_b_ (green) strands form the S-shaped excursion. Turn regions T_1_ through T_5_ (light gray rounded arrows) connect the β-strands. In some cases, turn T_3_ is replaced with an extended Ω-loop (21). Also indicated are the FxxN (red bold letters), GGA (I, V, L) (blue bold letters) motifs, and the 11 rung positions, which have side chains that always point inward, designated as *a* through *k* (light grey letters). (**B**) Four examples show the similar packing of non-canonical FxxN (red) and GGA (I, V, L) (blue) rungs, despite sequence differences. The fragments shown include *C. trachomatis* PmpB *Rungs 21*, *16*, *11,* and *4* (residues 316–330: 515–529, 737–751, and 1021–1034, respectively). (**C**) Summary table of repeat-region rungs, here for the predicted PmpE structure. Included for each rung are the rung number, the positions of the FxxN and GGA (I, V,L) motifs, and the length of the Ω-loop. A ribbon model of the *Ct*PmpE passenger domain is shown to the right of the table.

The β-helical repeat region of the passenger domain consists of a stack of highly regular rungs (Fig. 5A). Each of these rungs are composed of at least 27 residues that form five β-strands alternating with five turns (T) and have the general structure β1-T1-β2-T2-β2a-T3-β2b-T4-β3-T5 (Fig. 5A). While the T3 turn is often a tight turn composed of 3–5 amino acids, it can also contain an extended loop that varies greatly in length and constituency. These extended T3 loops, termed Ω-loops (21), always begin and end with a pair of FxxN and GGA (I, V, L) motifs that pack against each other to form a stable base. Each rung initiates with strand β1, which we defined as the first strand of the rung because the rung-to-rung transitions regularly occur in the preceding turn (i.e., T5) (Fig. S4A). The three longer β-strands of each rung – β1, β2, and β3 – form a core triangle with a well-defined hydrophobic interior. This triangle is interrupted by an S-shaped excursion that includes a turn (T2), formed by the xxN residues of each FxxN, the β2a strand leading into either a tight T3 turn or an Ω-loop of variable length, followed by the short β2b strand that guides the sequence back into the final turn (T4), which consists of the GGA (I, V, L) motif sequence (Fig. 5A; Fig. S3). The β3 strand completes the rung and T5 transitions to the next rung. The predicted regularity of these helical structures is evident in an end-on view of the rungs of the PmpE repeat region β-helix (Fig. S4B). The side chains of 11 residues within each rung (labeled ***a*** through ***k*** in Fig. 5A) always point into the β-helix and contribute to the hydrophobic interior of the rung structure. This includes the conserved residues in each of the tetrapeptide motifs.

We then looked more closely at the FxxN and GGA (I, V, L) motifs, exploring their positioning and possible function as key elements. The motifs FxxN and GGA (I, V, L) are centered on the T2 and T4 turns, respectively, in each rung of the helix (Fig. 5A). The motifs in each rung interact with each other, with the conserved residues of each FxxN packing against the GGA (I, V, L) motif that is upstream relative to helical assembly (since it folds in the C- to N-terminal direction) but downstream relative to the sequence (Fig. S4).

In addition to the tight spatial packing of these groups within and between rungs, the conserved Asn residues of the FxxN motifs form an “asparagine ladder” (Fig. S4C), similar to those seen in other β-helices (13). Within this ladder, each Asn side chain participates in four hydrogen-bonds: two to backbone atoms on each rung, helping stabilize the T2 conformation, and one each to the Asn residues in the rung above and the rung below. This ladder is capped by the conserved Asp discussed above, which forms key hydrogen bonds that stabilize the T2 turn in the first rung (Fig. S4C). This transition from the Pmp middle region to the repeat region in the passenger domain is highly conserved. In an alignment of all 123 Pmps analyzed in Fig. 3, all contained the conserved “D” in the same *Rung 0* location (Fig. S4D).

#### Ω-loops protrude from a common side of the passenger domain

As noted above, the Pmp repeat domain includes variable length loops that extend off from one surface of the β-helix and have no confidently predicted structure (Fig. 2, 4, and 5C; Fig. S1). This same surface is also the side from which the unstructured loops in the Pmp middle region protrude, so the full set of unstructured loops protrudes from a common planar surface of the protein. In a previous study (17), we determined that a set of these loops, termed VR1–VR5 (Fig. 1) were correlated with tissue tropism of different *C. trachomatis* strains. Recognizing the potential functional importance of these loops, we have developed a simple rung-by-rung tabular summary of the length of each Ω-loop and its corresponding framing FxxN and GGA (I, V, L) pair (Fig. 5C; Fig. S5). The Ω-loops found in the Pmp middle region (e.g., VR3, 4, and 5 in PmpE; Fig. 1B and 2) lack the tetrapeptide motif pairs found in the repeat region and are not included in the tables. However, these loops and the loops in the repeat region are modeled to be present on a common face of the β-helix.

#### Non-canonical tetrapeptide motifs are found in many *Chlamydia* spp. Pmps

**Fig 6:**
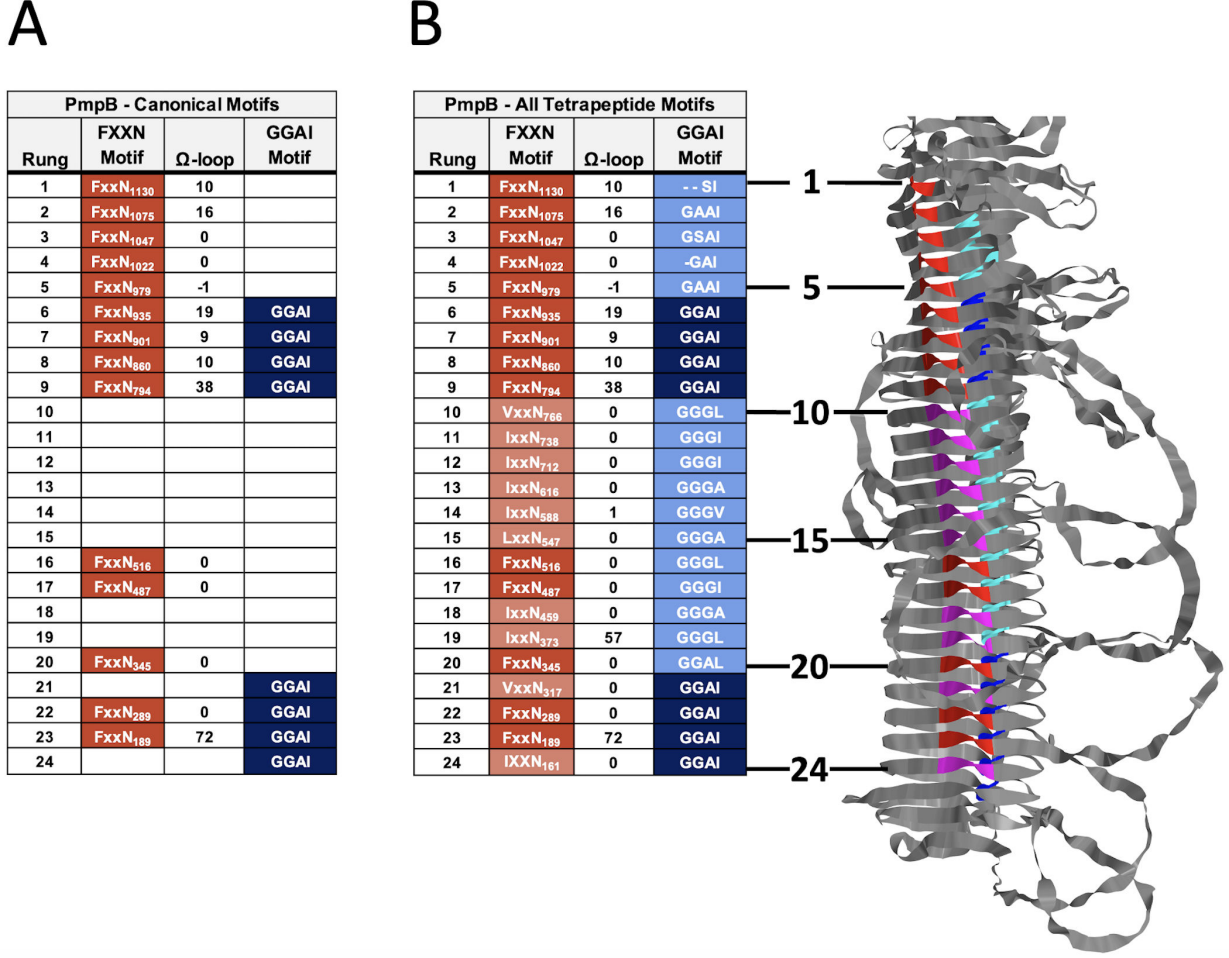
Predicted *C. trachomatis* Pmp structures include non-canonical tetrapeptide motifs serving the same structural role as canonical motifs. (**A**) Table of rungs for AlphaFold2 predicted model of PmpB including only canonical FxxN (red) and GGA (I, V, L) motifs. (**B**) A similar table of rungs as in panel A but including non-canonical FxxN (pink) and GGA (I, V, L) (cyan) motifs. A ribbon model of the PmpB repeat region is shown at right, with motif positioning and color coding identical to that shown in the tables.

PmpE has canonical motifs present in each rung of its repeat region (Fig. 5C). However, in some Pmps, the standard rung structure is predicted for all rungs in the repeat region, but many of the rungs lack one or both canonical tetrapeptide motifs. *C. trachomatis* PmpB, for example, has several consecutive rungs in the repeat region that lack the standard tetrapeptide motif pattern shown for PmpE but retain the β-helical structure of the region (Fig. 6). We hypothesized that variant motifs might be present in these rungs, satisfying the structural requirement for forming the rungs of the repeat region. This was shown to be true for every PmpB rung that lacked one, the other, or both canonical tetrapeptide motifs, as similar “noncanonical” motifs are found that supply the same structural features of canonical motifs (Fig. 5B and 6). Examples of noncanonical motifs in PmpB include GGGI or GGVI in place of GGA (I, V, L), and IxxN, LxxN, or VxxN in place of FxxN (Fig. 6). Furthermore, this observation holds true for every *C. trachomatis* Pmp (Fig. S5), as in each rung of the repeat region, either the canonical tetrapeptide motifs or structurally similar non-canonical ones are present, leading to a consistent rung structure throughout the helix. Examples of this are also found across the genus, as shown for one of the longest family members, *C. pneumoniae* Pmp20 (Fig. S5I).

#### Similar tetrapeptide motif-containing proteins outside of *Chlamydia* spp

To find any experimentally determined protein structures bearing notable similarity to the Pmp passenger domains, we used DALI (22) to compare the predicted structure of *Ct*PmpE with structures in the Protein Data Bank (23). The most structurally similar proteins were not close matches, but simply had generic β-helical rungs with a triangular cross section (Fig. S6). We conclude that the specific folding patterns of both the globular β-helix formed by the Pmp middle region and the regular β-helix with its S-shaped excursions formed by the Pmp repeat region are novel.

**Fig 7:**
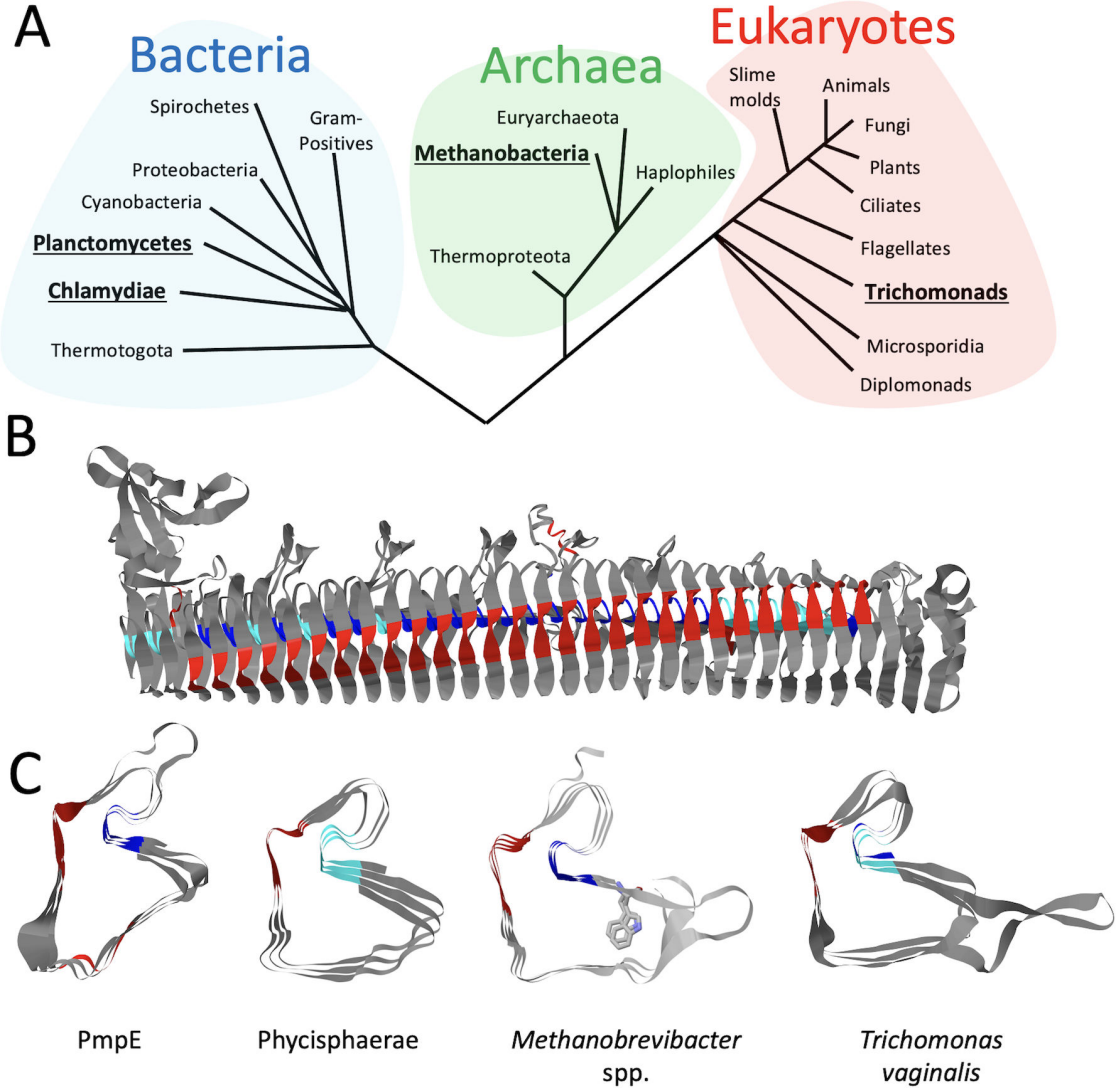
FxxN and GGA (I, V, L) motif-containing proteins occur in predicted β-helices from diverse organisms. (**A**) Phylogenetic tree with representative groups from the bacterial, archaeal, and eukaryotic domains, highlighting a non-chlamydial group in each domain (underlined and in bold) that has a protein with repeating FxxN and GGA (I, V, L) motifs predicted to be in a β-helix. (**B**) AlphaFold2 predicted the β-helical structure of residues 1–1,206 of the *Methanobrevibacter* Myelin-associated glycoprotein (MAG): Ig-like domain repeat protein (GenBank ID: MBQ2830912.1). The FxxN (red) and GGA (I, V, L) (blue) motifs are highlighted. (**C**) View down the β-helix axis of three rungs each from *Ct*PmpE (residues 156–246), a *Phycisphaerae* MAG: hypothetical protein (residues 218–295; GenBank ID: MBL7186538.1), the *Methanobrevibacter* protein shown in panel B (residues 445–542), and the *Trichomonas vaginalis* putative outer membrane protein (residues 127–219; GenBank ID: EAY21870.1). In each of the predicted structures, the FxxN (red) and GGA (I, V, L) (blue) motifs adopt equivalent local structures in each rung, while other parts of the rung are structurally different.

Because of the critical roles identified for Pmp tetrapeptide motifs in the structure of each Pmp β-helix, we then asked if such motifs might be present and serve a similar function in proteins from other species. This was initiated with a search of the literature and global protein sequence database for proteins carrying the signature tetrapeptide motifs. Work by other investigators identified the Pmp-signature motifs in the eukaryote *Trichomonas* spp. (24) and a Pfam family (PF02415) was identified that contains many proteins from over 100 non-chlamydial species that encode a *Chlamydia* Pmp-like β-helical repeat region (https://www.ebi.ac.uk/interpro/entry/pfam/PF02415/). Blast searches also identified similar motifs in the bacterium *Phycisphaerae* and the archaeon *Methanobrevibacter* spp. In the *Methanobrevibacter* spp. protein the canonical and non-canonical motifs are similar to those observed in the *Ct*Pmps, and in the *Phycisphaerae* protein, while the FxxNs remain the same, these are often paired with GGGM non-canonical motifs, a pattern not observed in the *Ct*Pmps (Fig. 7; Fig. S7). In each of these proteins, the FxxN and GGA (I, V, L) motifs are predicted by AlphaFold2 to be similarly placed and packed together to stabilize β-helical rungs. A comparison of these β-helices with those predicted for *Chlamydia* spp. Pmps showed that while the motifs have similar structural functions, the β-helical rungs in the various proteins have different overall shapes (Fig. 7; Fig. S7).

## DISCUSSION

The expanded family of chlamydial Pmps are attractive candidates for vaccine targets because of their surface localization, abundance, and potential function in target cell binding and tissue/host tropism. Members of this family have undergone diversification via orthologous and paralogous pathways (16), but there remains sequence and structural conservation across the genus (Fig. 3; Fig. S2). We used AlphaFold as a tool to model Pmp structure, with a particular focus on the localization and predicted function of paired tetrapeptide motifs that have been identified and explored by other investigators [reviewed in (16)]. Our analysis demonstrates that these motifs occur in highly regular patterns across all *C. trachomatis* Pmps and in each Pmp from other species that we have examined (Figs S2, S5I, and S7). Framed by these motifs are sets of variable loops, termed Ω-loops, that extend away from a common face of each Pmp (Fig. S5) and are tropism-predicting in *Ct*PmpE. The exposure of the disordered Ω-loops at the bacterial surface leads to a hypothesis that they may, in general, be critical points of contact between the bacterium and important ligands during infection and intracellular growth. In contrast, amino acid side chains of the FxxN and GGA (I, V, L) tetrapeptide motifs found within each chlamydial Pmp are predicted to be buried in the β-helical passenger domain (Fig. 5A). This placement reduces their availability to function directly in protein:protein interactions during chlamydial infection and growth and also reduces their opportunity to serve as neutralizing targets in immunized hosts.

AlphaFold has consistently been shown to predict protein structures within about 2 Å of the correct structure with the topology and broad structural features present (20, 25, 26). Thus, the high pLDDT values of the Pmp core regions lend confidence to our AlphaFold-based predictions of each Pmp, especially with regards to the overall structure of the β-helical N-terminal passenger domain (Fig. 2). In contrast, there is little confidence in the predicted internal structure of any Ω-loop within these Pmps, as is reflected by their lower pLDDT values (Fig. 2) for each loop in each Pmp. It has been proposed that quick and significant drops in pLDDT value, such as these, signify an intrinsically disordered region of the protein (27) that could adopt a more folded structure with either host or bacterial ligands at the chlamydial cell surface.

We hypothesize that the β-helical structure facilitated by the tetrapeptide motif pairs in each Pmp provides a consistent and stable platform that positions a set of Ω-loops that are (i) highly accessible at the bacterial cell surface, and (ii) do not specifically contribute to the folding of the protein. Thus, they are available to function in host cell attachment and infection, and their modification through evolutionary processes and/or laboratory-based excision or replacement could alter tissue tropism without disrupting the overall Pmp structure. This hypothesis is supported by our previous analysis of tropism-predicting sequences in *C. trachomatis* PmpE (17). These studies identified a set of tropism-predicting variable regions in PmpE (VR1–VR5) which correspond to Ω-loops in *Rungs 8, 1, –3, −4,* and *−5,* respectively, in the protein (Fig. 1B, 4A and 5C). For purposes of protein engineering, the margins of Ω-loops in the Pmp repeat region are consistently bracketed by the β2a and β2b strands, providing a precise guide for specific epitope replacement using molecular genetic approaches. Specifically, the common structure of a tight T3 turn (Fig. 4A; Fig. S3B), implies that the splice point for an Ω-loop is between the residues three positions after the N of the FxxN and the residue two positions before the first G of the GGA (I, V, L). In contrast, Ω-loops in the middle region are framed by the β3 and β1 sheets, with less predictability as to the exact entry and exit points of the loops (Fig. 4). Despite the difference in rung structure between these two regions, the Ω-loops remain in a consistent position extending out from the β-helix on a common predicted face of the molecule, collectively available for interacting with specific protein targets.

As noted above, autotransporter proteins are secreted starting with the C-terminal end of the extracellular passenger domain, and for this reason their passenger domains fold from the C- to the N- terminus, backward from the direction of protein folding coming off of the ribosome. For many autotransporters, this directionality has been shown to be important, in that a C-terminal portion of the passenger domain serves as an “autochaperone” that is required for proper folding of the N-terminal parts of the passenger domain (28). With this in mind, something striking about the Pmp proteins is the greater globularity of the Pmp middle region (with its C-terminal cap of two long antiparallel β-strands and *Rungs −5* through *Rung 0*) is consistent with the region having a strong driving force for folding properly. It is possible that the domain could then be responsible for helping promote the correct folding of the more regular, but less globular repeat region of the passenger domain. The expectation that the Pmp middle region folds first is why we refer to the key aspartic acid found in all Pmps as a ‘seed’ rather than a ‘cap’ for the asparagine ladder. We further note that if proper folding of the Pmp proteins is required for infectivity, learning how Pmp proteins fold and how that folding can be disrupted can provide a novel strategy for developing therapeutics.

The identification of the canonical tetrapeptide motifs in Pmps was reported in the description of the C. *trachomatis* genome (14). Subsequent genome sequencing has identified the motifs in each examined chlamydial Pmp protein (16). Our AlphaFold-based analysis demonstrates a general pattern of motif pairing, and adds the important observation that non-canonical motifs fill apparent gaps such that every rung in the repeat region has a pair of motifs, whether canonical or not. Furthermore, such motifs are not exclusive to *Chlamydia* or even limited to bacterial systems. FxxN and GGA (I, V, L) motifs or their variants are present in β-helix containing domains in various species, where they display consistent positioning relative to their location in the Pmps (Fig. 7; Fig. S7). In examined proteins outside of *Chlamydia*, while the S-shaped excursion created by the interacting motifs is similar, there are few other similarities that exist in sequence or rung structure.

Our work offers a broader perspective of the Pmp family and suggests the need for recontextualizing some previous analyses regarding the proteins and especially their motifs. As one example, a study by Favaroni and Hegemann (29) reported that select recombinant *C. trachomatis* Pmp fragments formed functional adhesion-competent multimers that assembled into homo- and heteromeric filaments. Our work shows that these fragments are composed of a mixture of Pmp middle regions and repeat regions that, respectively, lack regions of the protein crucial for folding. The formation of multimers could then be due to the fragments lack of structural integrity leading to aggregation rather than being a true representation of how these proteins interact *in vivo*.

Our study provides a structural foundation to guide more in-depth analysis of the chlamydial Pmp family. Leveraging the predictive power of Alphafold2, we observed a high degree of structural similarity across Pmp proteins and illuminated their common structural features. The characteristic FxxN and GGA (I, V, L) tetrapeptide motifs and their non-canonical counterparts are integral parts of the β-helix structure in all examined chlamydial Pmps, as well as in other species. Also, highly accessible Ω-loops, framed by motifs in the repeat region, protrude from the bacterial surface where we hypothesize they can mediate host cell attachment and/or facilitate infection. In contrast, the tetrapeptide motifs are buried within the β-helical passenger domain, making them less available for direct protein:protein interactions during infection. This, in combination with the presence of motifs outside of *Chlamydia*, challenges their previously hypothesized role as adhesion sites. The descriptive framework of the structure of Pmp proteins provided here will help guide laboratory-based mutational studies to dissect the roles of these Ω-loops and provide valuable insight into how these proteins function and a basis for new therapeutic strategies.

## MATERIALS AND METHODS

### AlphaFold2-based structure predictions

Protein structures were predicted using the AlphaFold2 AI protein structure prediction system. The Pmps of *C. trachomatis* serovar D/UW/3 are web-accessible via the online AlphaFold protein repository (20; https://alphafold.ebi.ac.uk/). The Uniprot numbers giving access to each of these nine predicted structures are: *Ct*PmpA—O84417, *Ct*PmpB—O84418, *Ct*PmpC—O84419, *Ct*PmpD—O84818, *Ct*PmpE—O84877, *Ct*PmpF—P38008, *Ct*PmpG—O84879, *Ct*PmpH—O84880, and *Ct*PmpI—O84882. For predicting the structure of proteins not available in the AlphaFold repository, we used ColabFold v1.5.2 to run AlphaFold2 using MMseqs2 (25). Proteins shorter than 1,400 residues were predicted in their entirety; those that exceeded 1,400 amino acids were predicted in adjacent chunks of 1,200 amino acids. AlphaFold2 produces and ranks five models for each protein it predicts, along with the pLDDT, predicted aligned error, and sequence coverage for each position in each model. For all predictions we carried out, the five models were all similar, and we chose the model with the highest overall pLDDT. Predicted protein structures were viewed and annotated using the Geneious protein viewer (30), and some figures were made using Pymol (https://pymol.org/2/support.html).

### Protein sequence analyses

The signal peptide cleavage site for each Pmp was predicted using the Signal P 5.0 webserver (31). For generating a multiple sequence alignment of all chlamydial Pmp proteins, we included all Pmp proteins listed in Fig. 1 of (16) and filtered out 11 sequences that did not contain both an autotransporter and a passenger domain (*C. caviae*: PmpE-1; *C. felis*: Pmp02, Pmp03, Pmp06, Pmp14, and Pmp15; *C. gallinacea*: PmpF and PmpI *C. pneumoniae*: Pmp12; *C. psittaci*: Pmp5 and Pmp18), and four sequences that were <500 residues long (*C. abortus*: Pmp5E; *C. caviae*: PmpE-2; *C. pecorum*: PmpE2; *C. psittaci*: Pmp17). Pmp4 and Pmp5 from *C. psittaci* have had their schematic representations swapped, hence why Pmp5 was excluded. The remaining 123 sequences were downloaded from the NCBI protein database and then aligned using the Clustal Omega Web server (32) program Clustalo v1.2.4. Phylogenetic trees were created using Geneious v11.0.15 + 10 (30) from the Clustal Omega Web server alignment. Trees were neighbor-joining consensus trees with an outgroup of the *Helicobacter pylori* protein VacA that was forced after the tree was run. An unrooted tree can be seen in Fig. 2, and a rooted version can be found in Fig. S2. In both cases, branches were not transformed.

### Discovery of β-helices containing paired canonical or non-canonical tetrapeptide motifs in proteins outside of *Chlamydia* spp

The first non-Pmp having FxxN and GGA (I, V, L) motifs, we considered was a protein in *Trichomonas vaginalis* identified by Handrich et al. (24). A BLAST search of this protein, excluding hits from *Chlamydia* identified a Phycisphaerae bacterium protein and a *Methanobrevibacter* spp. protein (Fig. 7). Additionally, PFAM has a repository of *Chlamydia* Pmp repeat domains (PF02415) [https://www.ebi.ac.uk/interpro/entry/pfam/PF02415/; (33)], which contains a number of proteins not from the *Chlamydia* genus. A selection of proteins from this list that also contained FxxN and GGA (I, V, L) repeats were selected for AlphaFold2 modeling and comparison as well (Fig. S7). A search of the PDB90 subset of protein structures was conducted using DALI (22); Fig. S6]. Segments of *Ct*PmpE (*Rungs 5–7*, repeat region, and middle region) were put into the DALI web server and we examined the most similar hit for each segment.

## SUPPLEMENTAL MATERIAL

Supplemental material spectrum.03242-23-s0001.pdf Table S1 and Fig. S1 to S7.

ASM does not own the copyrights to Supplemental Material that may be linked to, or accessed through, an article. The authors have granted ASM a non-exclusive, world-wide license to publish the Supplemental Material files. Please contact the corresponding author directly for reuse.
