# Supplementary material for: A structural foundation for studying chlamydial polymorphic membrane proteins
Source: Microbiol Spectr. 2023 Oct 26;11(6):e03242-23. doi: 10.1128/spectrum.03242-23 (PMC10715098; doi:10.1128/spectrum.03242-23)
Supplement: Supplemental material — Table S1 and Fig. S1 to S7. [file spectrum.03242-23-s0001.pdf]

Supplemental Figures, Debrine *et al.*

Table S1. Pairwise percent amino acid sequence identities for the nine *C. trachomatis* D/UW/3 Pmp proteins. The upper right half of the matrix shows pairwise amino acid identities for each intact Pmp. The lower left half shows pairwise identities for the passenger domain only. The alignments on which these identities were based were conducted using Clustal Omega (1; https://www.ebi.ac.uk/Tools/msa/clustalo/), with no subsequent manual editing. The Uniprot IDs for the nine proteins are: PmpA - O84417, PmpB - O84418, PmpC - O84419, PmpD - O84818, PmpE - O84877, PmpF - P38008, PmpG - O84879, PmpH - O84880, PmpI - O84882.

Figure S1. AlphaFold2 predicted models of the nine *C. trachomatis* D/UW3 Pmp proteins. Ribbon diagrams of each protein are shown using the same scale and with canonical FxxN (red) and GGA(I,V,L) (blue) motifs, and non-canonical FxxN (magenta) and GGA(I,V,L) (cyan) motifs highlighted. Uniprot IDs for the nine proteins are: PmpA - O84417, PmpB - O84418, PmpC - O84419, PmpD - O84818, PmpE - O84877, PmpF - P38008, PmpG - O84879, PmpH - O84880, PmpI - O84882.

Figure S2. A full phylogenetic tree of Pmp proteins from 10 *Chlamydia* spp. A full neighbor-joining rooted phylogenetic tree is shown without distance corrections. This tree is built with the same Pmps shown in Fig. 2. The tree is rooted with an autotransporter protein from *Heliobacter pylori* (accession number: P55981.1), a member of autotransporter phylogenetic group 8 (2). The scale bar represents the estimated number of substitutions per site. Boxed in purple are the *Ct*Pmps. An equivalent comparison of the same proteins but with the autotransporter domain removed yielded a similar tree, with all proteins having at least one *Ct*Pmp with which they shared a clade.

Figure S3. Comparison of the PmpE repeat region topology (A) with the amino acid sequence (B). A. Topology model as shown in Fig 4A. **B.** The amino acid sequence of the PmpE passenger domain organized to show amino acid placement in the topology diagram. In both images, the N-terminus is near the bottom, and the C-terminus of the passenger domain is near the top. Highlighted are the division between the middle and repeat-containing region (dotted line), line rung numbers, residue numbers that initiate each rung and end the domain, β-strands (colors as in panel A), helices (underlined), and lengths of Ω-loops in place of amino acid sequence. Bold violet numbering is used for the Ω-loops corresponding to VR1 – VR5 regions that correlate with tissue tropism (3). The letters ***a* - *k*** listed below *Rung 9* identify the positions with side chains pointing inwards in each rung of the regular repeat portion of the domain (see Figure 5A).

Figure S4. Detailed presentation of the repeat region rung structure in the predicted structure of all of *Ct*Pmps A. *Rungs 1* through *4* of PmpE with β-strand coloring as in Fig. 5A. The T_5 turn is positioned at the front. In contrast to Figures 1B and 4B, the β-helix is rotated 180˚ around the helix axis, positioning the T_3 turns and Ω-loops on the left and the β2-T_2 segment, containing the FxxN motif, is positioned at the back right to left. Noteworthy in this image is that the β-helix does not rise like a corkscrew, but instead, has a step-like rise between rungs that largely occurs within the T_5 turn. This image also demonstrates that within each rung, FxxN (starting at the end of β2) is at the same level as the following GGA(I,V,L) motif (ending at the start of β3). **B.** A stick model of 8 rungs of the repeat region β-helix axis of *Ct*PmpE (residues 80-340, from *Rung 9* to *Rung 2*) viewed from the amino-terminal end of the structure. This image shows the consistent positioning of the side chains of the nine inward facing residue positions (labeled *a,b,c,d,e,f,i,j,k*) and consistency in overall rung structure. The FxxN (red) and GGA(I,V,L) motifs are highlighted. **C.** *Rung 0* through *Rung 3* of PmpE using the same view as in Panel A, but with the front section cut away to reveal the β2-T_2 FxxN side chains in the helix core (labeled in *Rung 2*). Highlighted are hydrogen bonds (H-bonds) that build the β2 and β3 sheets (yellow dashed lines) and those involving the Asn of the FxxN motif and the conserved Asp side chain from *Rung 0* (cyan dashed lines). The Asn side chains form an internal "asparagine ladder", with each Asn making four H-bonds, two that link it with the Asn residues on the previous and following rungs, and two that link it to backbone carbonyl and NH groups in the next Rung. At the top of the panel is the Asp side chain from *Rung 0* conserved in all chlamydial Pmp sequences which makes H-bonds with *Rung 1* residues to seed the overall

β-helix formation. Also labeled at the top left edge of the figure is the $\beta 2_b$ strand (green arrow with "$\beta 2_a$ seed" label) from *Rung 1* that makes a unique antiparallel sheet interaction with the $\beta 2_a$ strand from *Rung 1* in a way that appears to seed that sheet as the protein folds from the C- toward the N-terminus. These featured interactions are present in each of the predicted *Ct*Pmp structures.

Figure S5. **Models of chlamydial Pmp repeat regions with accompanying tables of motif and Ω-loops.** Alphafold2 predicted models of the *Ct*Pmps. **A.** Ribbon diagram for the predicted passenger domain structure of *Ct*PmpA from D/UW/3, adjacent to a summary table of the motifs and Ω-loops present in each the rung of its regular repeat portion. The information is as described in Figure 6, highlighting both canonical FxxN (red) and GGA(I,V,L) (blue) motifs and non-canonical FxxN (magenta) and GGA(I,V,L) (cyan) motifs. **B-H.** Each panel has equivalent information to panel A, but is focused on PmpB, C, D, F, G, H, and I, respectively. **I.** A similar representation for *C. pneumoniae* Pmp20.

Figure S6. Experimentally-determined structures most similar to segments of the *Ct*PmpE passenger domain. A. *Rungs 5-7* (residues 158-246) of *Ct*PmpE (green Cα trace) were overlayed with the top hit (orange Cα trace, thick for aligned residues and thin for unaligned residues) from a DALI search (4) of *Ct*PmpE *Rungs 5-7* against the "PDB90" representative set of Protein Data Bank (PDB; 5) structures. The top hit was PDB entry 4peu chain A described as a "lyase-like protein from *Clostridum thermocellum*" and the comparison statistics were: Z-score = 4.5, percent sequence identity = 15%, and 75 aligned residues out of 250. The view of the rungs is as in Figure 5A, and the β-strands β1, β2, and β3 of the *Ct*PmpE rungs are identified. **B.** The same as panel A but for a search using the entire *Ct*PmpE repeat region (residues 18-407). The top hit for this search was PDB entry 5ny0 chain A described as "SRRP binding Region from *Lactobacillus Reuteris*", and the comparison statistics were: Z-score =19.7, percent sequence identity = 12%, and 262 aligned residues out of 343.**C.** The same as panel A but for a search using the whole *Ct*PmpE middle region (residues 407-659). The top hit for this search was PDB entry 3sze chain A described as a "serine protease from *E. coli*", and the comparison statistics were: Z-score = 8.7, percent sequence identity = 12%, and 133 aligned residues out of 943. In addition to a view down the β-helix (left-hand image) as in panel A, an orthogonal view is shown (right hand image; similar to the view in Figure 1B) to clearly show how the best hit structure does possess the wider, more globular region that encompasses *Rungs -4, -5,* and the β-hairpin at the C-terminal end of the Pmp middle region.

Figure S7. Examples of proteins outside of chlamydia that contain paired FxxN and GGA(I,V,L) motifs. A. Alphafold2 predicted model of residues 0-430 I of a Phycisphaerae Myelin-associated glycoprotein (MAG): hypothetical protein (accession number: MBL7186538.1) and to the right a view down three rungs of its regular β-helix (residue numbers given in the figure) with canonical FxxN (red) and GGA(I,V,L) (blue) and non-canonical GGA(I,V,L) motifs (cyan) highlighted. **B-E.** The same as panel A but for the following: residues 0-280 of the *Trichomonas vaginalis* putative polymorphic outer membrane protein (POMP) (accession number: EAY21870.1) in panel B; residues 0-280 of the *E. coli* adhesin AidA-I_1 (accession number: A0A024L0S3) in panel C; residues 60-370 of the *Fischerella ambigua* POMP protein (accession: A0A076NA94) in panel D; and residues 74-213 of the *Saprolegnia parasitica* β- helix containing protein (accession number: A0A067CJ47) in panel E.

Table S1. Pairwise percent amino acid sequence identities for the nine *C. trachomatis* D/UW/3 Pmp proteins.

	PmpA	PmpB	PmpC	PmpD	PmpE	PmpF	PmpG	PmpH	PmpI
PmpA	-	20	18	19	22	19	21	25	22
PmpB	20	-	**50**	23	19	20	22	21	20
PmpC	18	**45**	-	22	19	19	20	19	18
PmpD	21	21	22	-	20	20	21	20	23
PmpE	23	23	21	22	-	**27**	22	23	23
PmpF	21	23	21	23	**28**	-	24	23	23
PmpG	24	23	21	25	24	21	-	24	**29**
PmpH	22	21	18	23	25	20	24	-	22
PmpI	23	23	21	22	26	23	**30**	24	-

Fig S1: AlphaFold2-predicted models of the nine *C. trachomatis* strain D/UW/3 Pmp proteins.

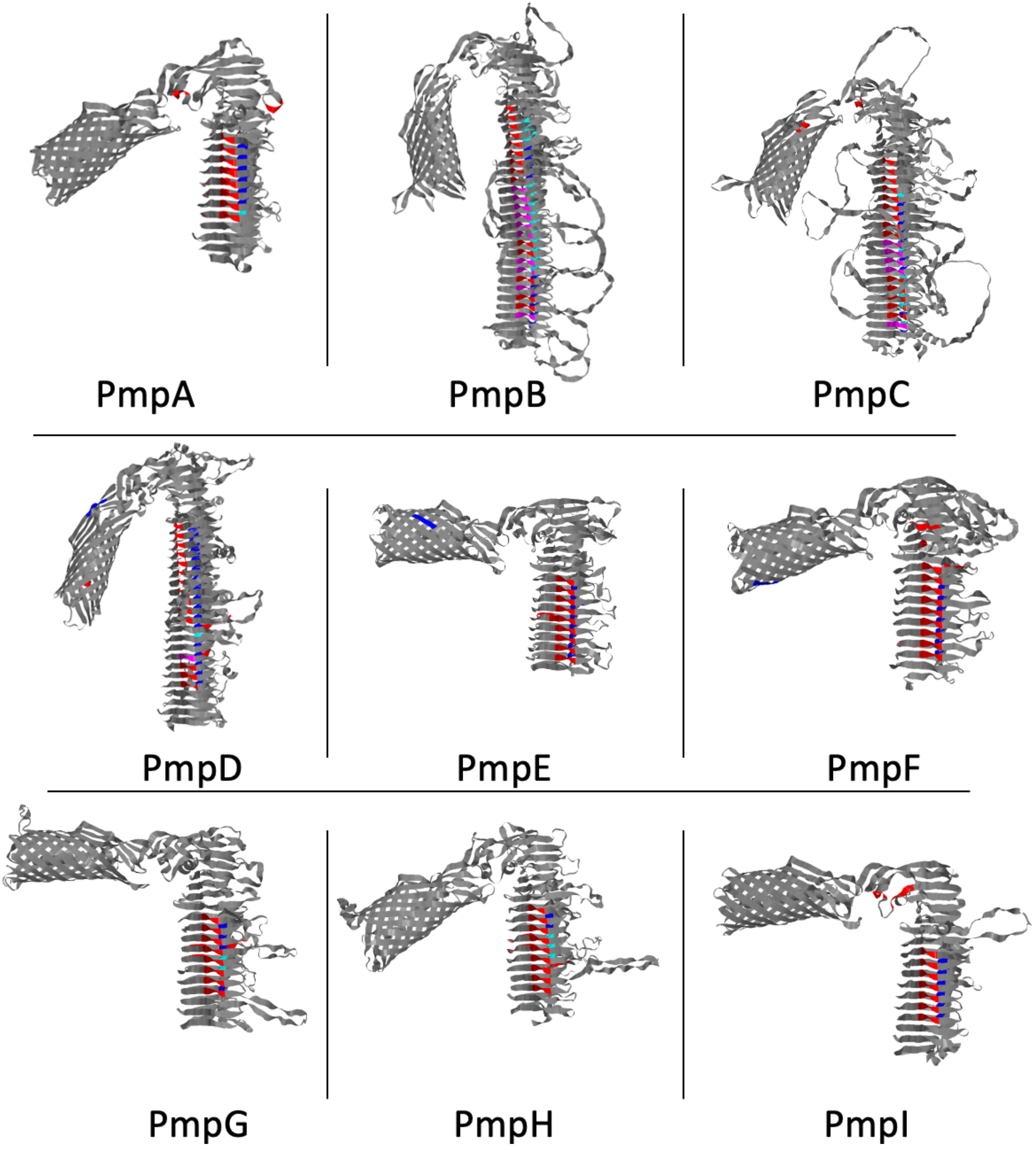

Fig S2: Phylogenetic tree of all Pmp proteins from 10 *Chlamydia* species.

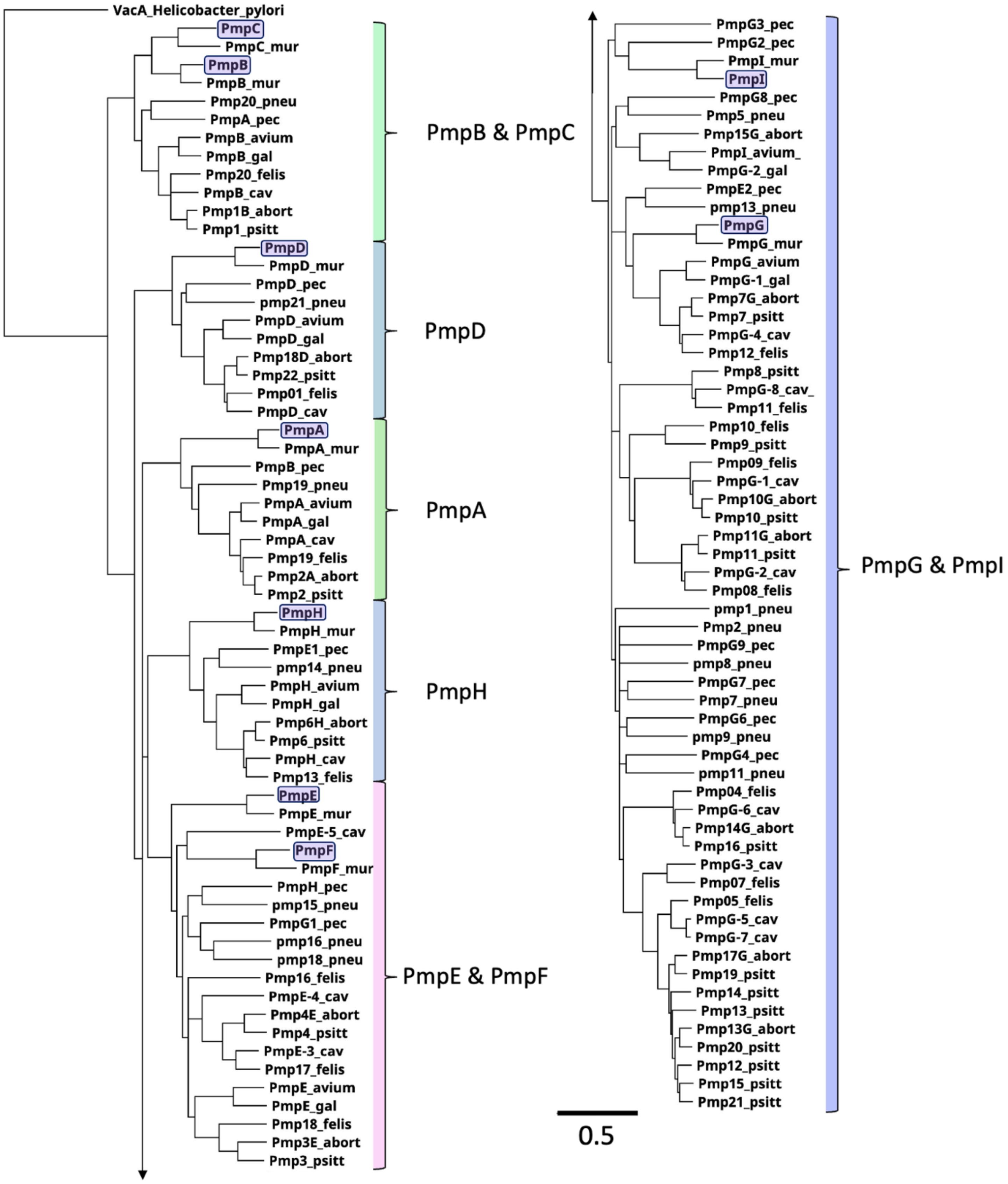

Fig S3: Comparison of the PmpE repeat region topology (A) with the amino acid sequence (B).

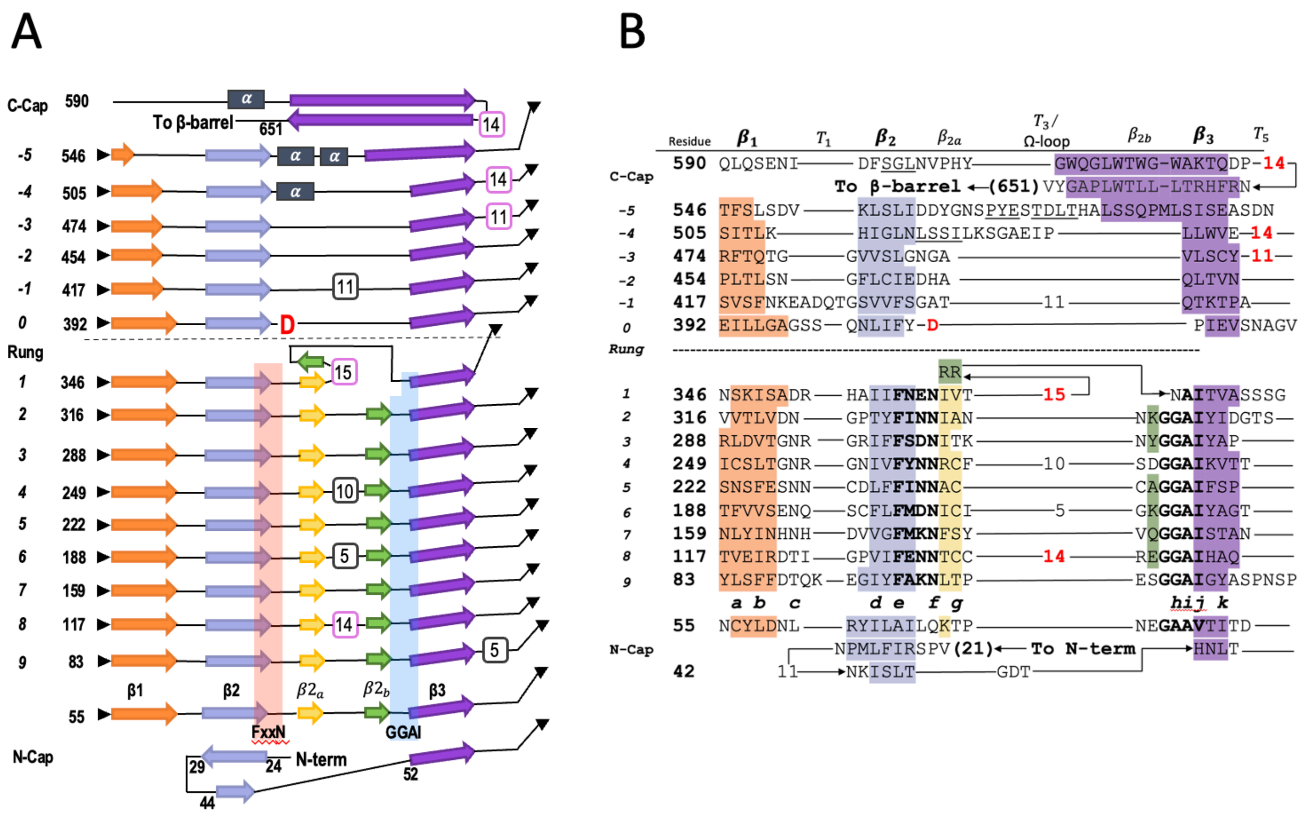

Fig S4. Detailed presentation of the repeat region rung structure in the predicted structure of all of *Ct*Pmps.

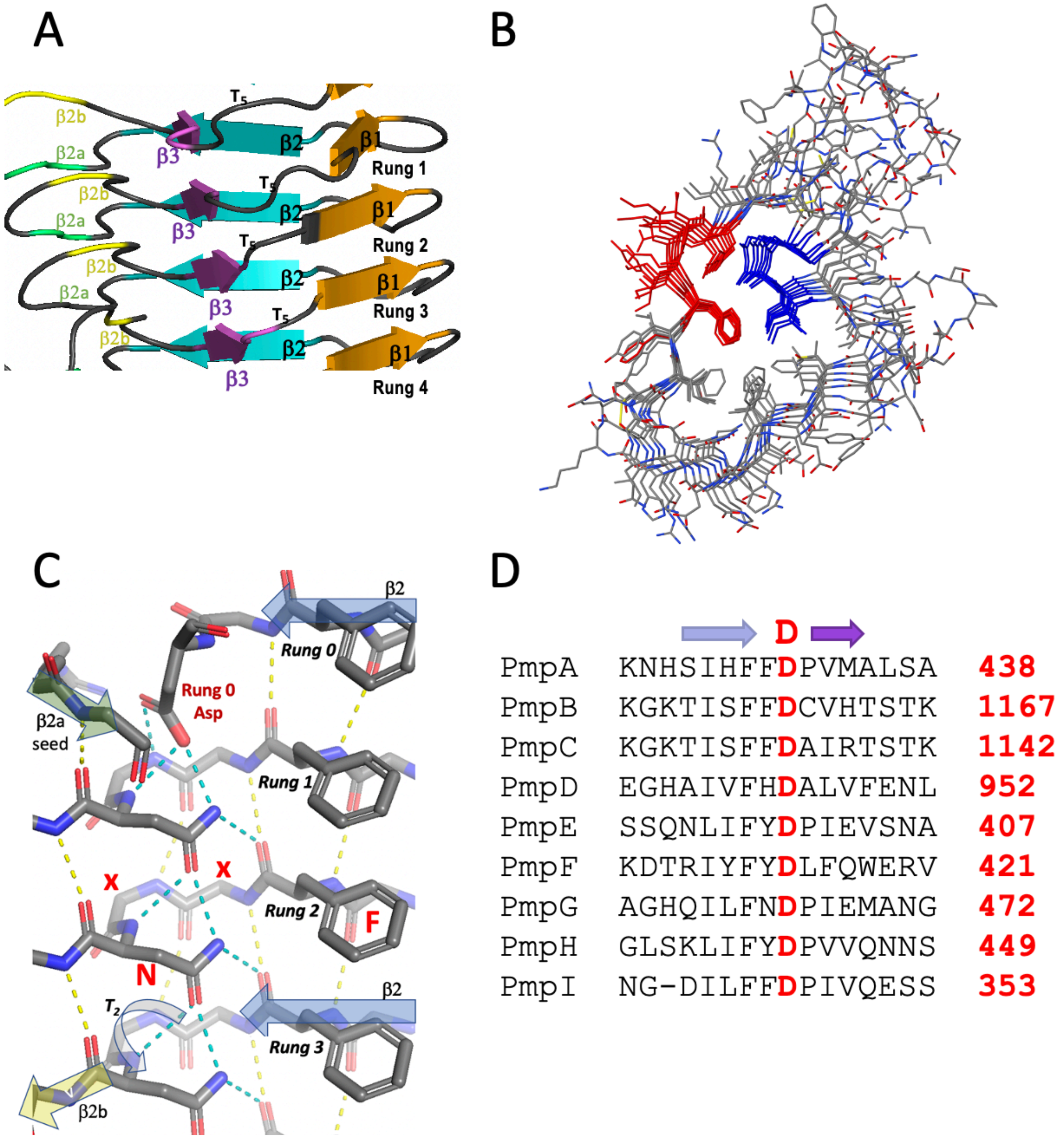

Fig S5: Models of chlamydial Pmp repeat regions with accompanying tables of motif and Ω-loops.

A

PmpA				
Rung	FXXN Type	N position	Ω-loop	GGAI Type
1	FxxN	398	8	- - SI
2	FxxN	365	6	GGAI
3	FxxN	338	5	GGAI
4	FxxN	310	5	GGAI
5	FxxN	282	5	GGAI
6	FxxN	253	4	GGAI
7	FxxN	214	13	GGAI
8	FxxN	181	13	GGVI

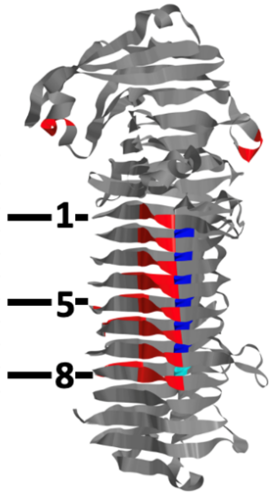

B

PmpB				
Rung	FXXN Type	N position	Ω-loop	GGAI Type
1	FxxN	1130	15	- - SI
2	FxxN	1075	21	GAAI
3	FxxN	1047	5	GSAI
4	FxxN	1022	5	-GAI
5	FxxN	979	4	GAAI
6	FxxN	935	24	GGAI
7	FxxN	901	14	GGAI
8	FxxN	860	15	GGAI
9	FxxN	794	43	GGAI
10	VxxN	766	5	GGGL
11	IxxN	738	5	GGGI
12	IxxN	712	5	GGGI
13	IxxN	616	5	GGGA
14	IxxN	588	6	GGGV
15	LxxN	547	5	GGGA
16	FxxN	516	5	GGGL
17	FxxN	487	5	GGGI
18	IxxN	459	5	GGGA
19	IxxN	373	62	GGGL
20	FxxN	345	5	GGAL
21	VxxN	317	5	GGAI
22	FxxN	289	5	GGAI
23	FxxN	189	77	GGAI
24	IXXN	161	5	GGAI

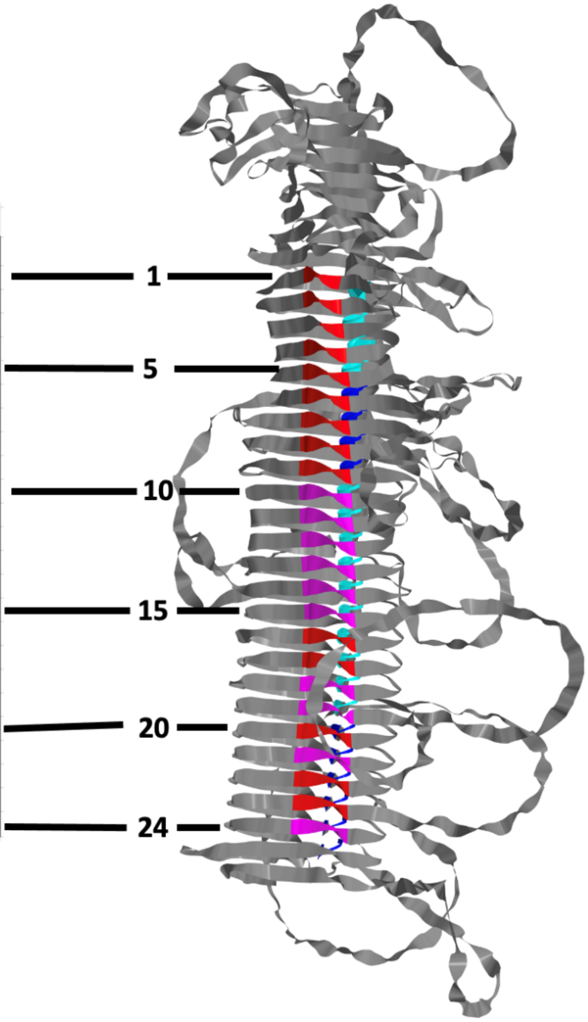

Fig S5, continued:

C

PmpC				
Rung	FXXN Type	N position	Ω-loop	GGAI Type
1	FxxN	1105	15	-- -- SI
2	FxxN	1046	24	GAAI
3	FxxN	1018	5	GSAI
4	FxxN	993	5	A--TI
5	FxxN	961	4	GSAI
6	FxxN	922	18	GGAI
7	FxxN	890	12	GGAI
8	FxxN	847	17	GGAV
9	FxxN	604	220	GGAI
10	SxxN	576	5	GGAI
11	LxxN	548	5	GGGL
12	IxxN	521	5	GGAI
13	LxxN	427	5	GGAI
14	FxxN	400	5	GGGI
15	FxxN	361	5	GGGA
16	FxxN	333	5	GAGV
17	FxxN	236	5	GGGI
18	FxxN	208	5	GGSV
19	VxxN	180	5	GGAI

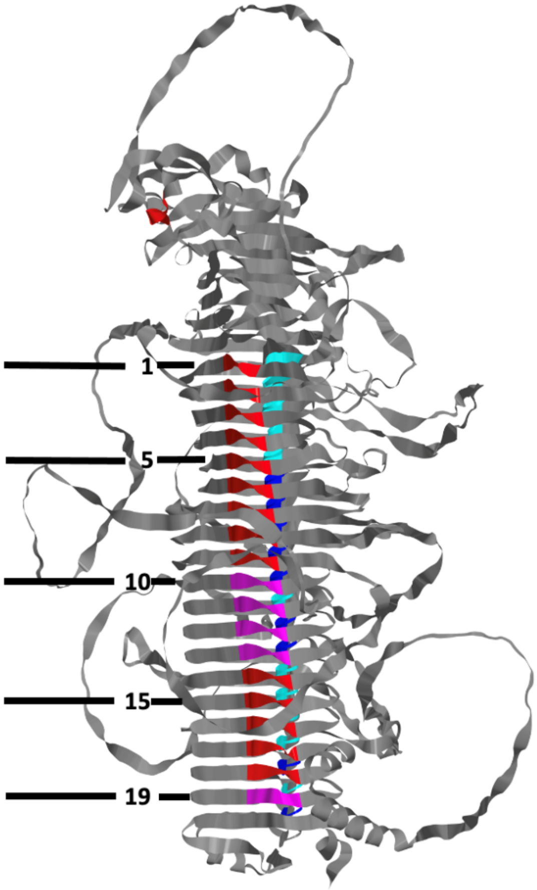

D

PmpD				
Rung	FXXN Type	N position	Ω-loop	GGAI Type
1	FxxN	917	9	-- -- AI
2	FxxN	887	4	GGAV
3	FxxN	852	12	GGAI
4	FxxN	811	5	GGAI
5	FxxN	715	73	GGAI
6	FxxN	685	5	GGAI
7	FxxN	656	5	GGAL
8	FxxN	626	8	GGAL
9	FxxN	587	16	GGAV
10	FxxN	535	30	GGAI
11	FxxN	498	14	GGAI
12	FxxT	461	13	GGAL
13	FxxG	422	4	GGGI
14	FxxS	393	5	GGAI
15	CxxN	365	5	GGAI
16	FxxN	330	12	GGAI
17	FxxN	303	4	GGAI
18	FxxN	273	6	GGAI

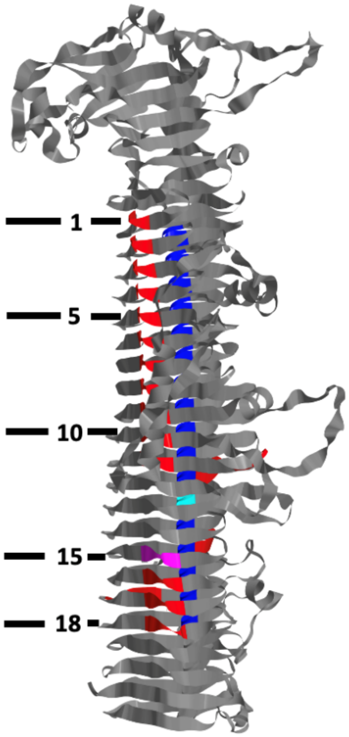

Fig S5, continued:

E

PmpF				
Rung	FXXN Type	N position	Ω-loop	GGAI Type
1	FxxN	379		
2	FxxN	347	4	--GAA
3	FxxN	320	5	GGAI
4	FxxN	283	13	GGAI
5	FxxN	256	4	GGAI
6	FxxN	215	16	GGAI
7	FxxN	186	5	GGAI
8	FxxN	146	18	GGAL
9	FxxN	116	7	GGAI

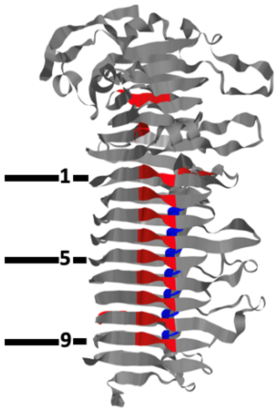

F

PmpG				
Rung	FXXN-Type	N position	Ω-loop	GGAI-Type
1	FxxN	427	20	-- -- AI
2	FxxN	397	4	GGAI
3	FxxN	370	5	GGAI
4	FxxN	310	26	GGAI
5	FxxN	283	4	GGGI
6	FxxN	275	5	GGGI
7	FxxN	240	5	GGA--
8	FxxN	183	5	GGAI

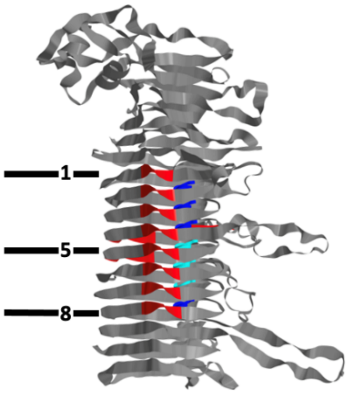

G

PmpH				
Rung	FXXN Type	N position	Ω-loop	GGAI Type
1	FxxN	414	11	-- -- IL
2	FxxN	379	4	GGAI
3	FxxN	351	5	GGAI
4	FxxN	324	5	GGSI
5	FxxN	276	23	GGVI
6	FxxN	248	5	GGVV
7	FxxN	142	39	GSI--
8	FxxN	115	6	GMI--

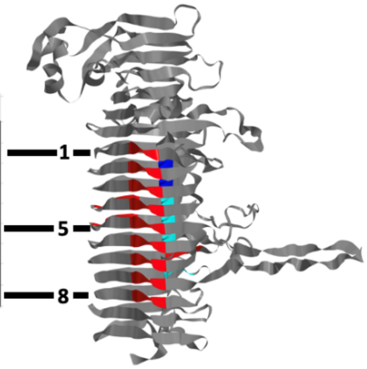

H

PmpI				
Rung	FXXN Type	N position	Ω-loop	GGAI Type
1	FxxN	317	12	-- -- AI
2	FxxN	287	4	GGAI
3	FxxN	260	5	GGAI
4	FxxN	225	4	GGAI
5	FxxN	197	5	GGAI
6	FxxN	169	5	GGAI
7	FxxN	141	5	GGAI

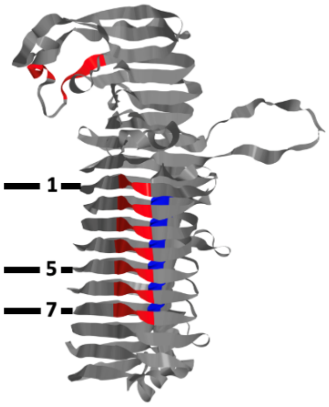

Fig S5, continued:

I

C. pneumoniae Pmp20				
Rung	FXXN Type	N position	Ω-loop	GGAI Type
1	FxxN	1149	6	-- -- IA
2	FxxN	1105	11	GAAI
3	FxxN	1077	0	GSAI
4	FxxN	1051	0	GGAI
5	FxxN	1016	-1	GGAI
6	FxxN	976	13	GGAI
7	FxxN	939	9	GGAI
8	IxxN	901	9	GGGI
9	IxxN	864	8	GGGI
10	VxxN	836	0	GGGI
11	VxxN	809	-1	GGGV
12	IxxN	782	0	GGGI
13	FxxN	749	0	GGAL
14	VxxN	722	0	GGGI
15	FxxN	676	2	GGGA
16	FxxN	648	0	GGGL
17	FxxN	619	0	GGGL
18	FxxN	591	0	GGGA
19	FxxN	523	39	GGAL
20	FxxN	495	0	GGAI
21	MxxN	466	0	GGAI
22	FxxN	438	0	GGGI
23	FxxN	396	13	GGAI
24	FxxN	313	55	GGAI
25	FxxN	284	0	GGGI
26	FxxN	256	0	GGAI
27	FxxN	227	0	GGAA

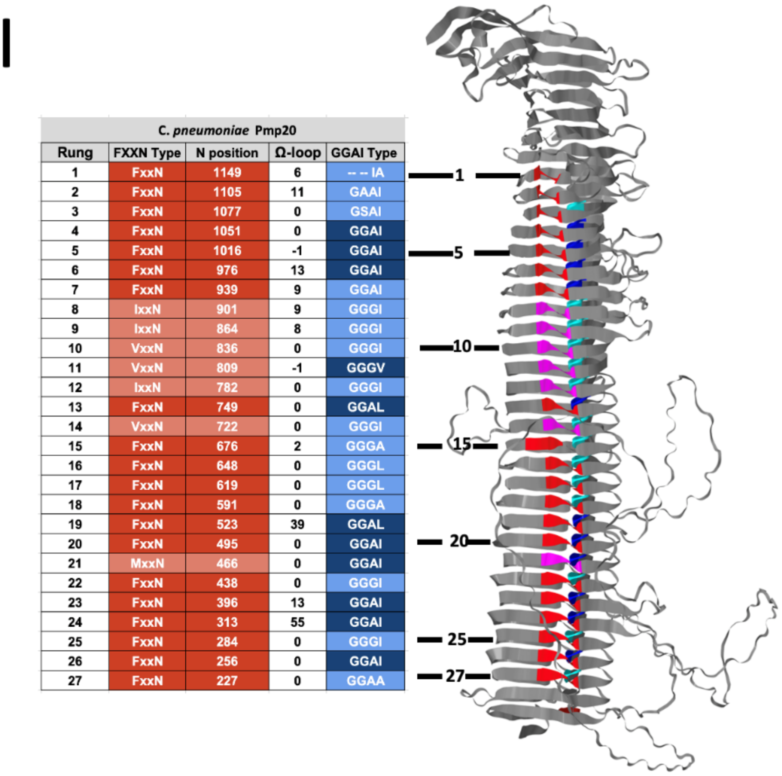

Fig S6: Experimentally-determined structures most similar to segments of the *Ct*PmpE passenger domain.

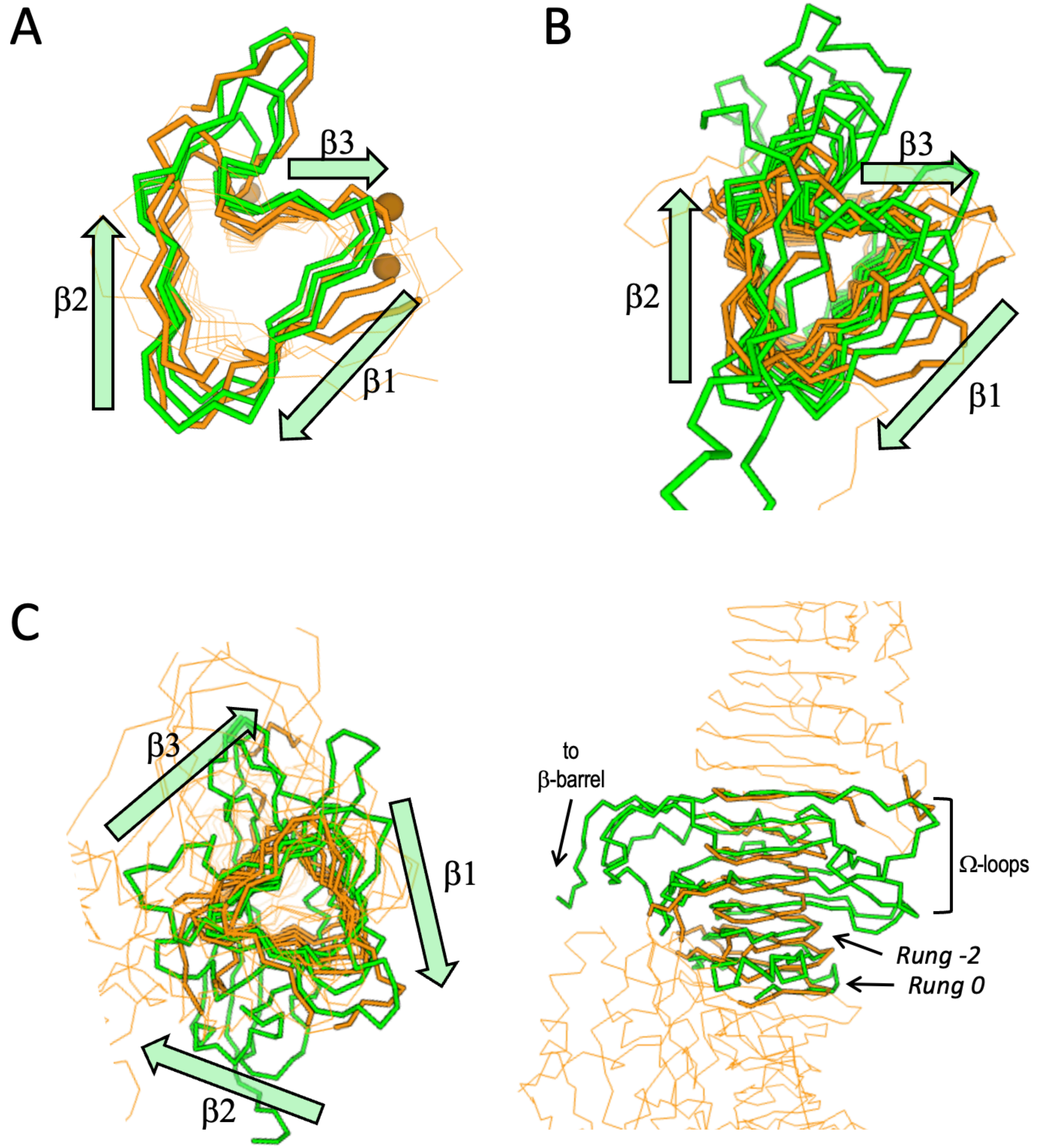

Fig S7: Examples of proteins outside of chlamydia that contain paired FxxN and GGA(I,V,L) motifs.

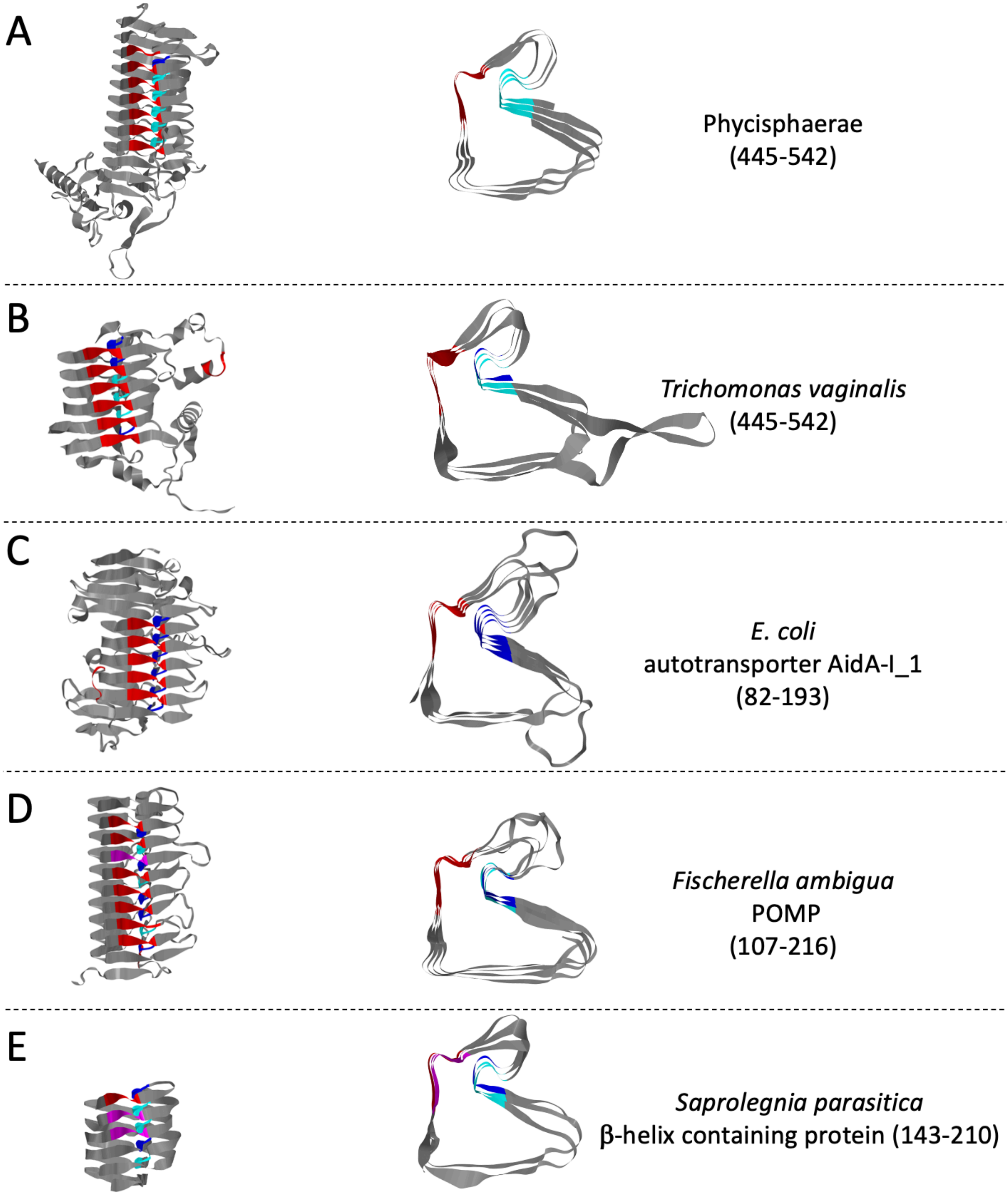

References

1. Sievers F, Wilm A, Dineen D, Gibson TJ, Karplus K, Li W, Lopez R, McWilliam H, Remmert M, Söding J, Thompson JD, Higgins DG. 2011. Fast, scalable generation of high-quality protein multiple sequence alignments using Clustal Omega. Mol Syst Biol 7:539.
2. Clarke KR, Hor L, Pilapitiya A, Luirink J, Paxman JJ, Heras B. 2022. Phylogenetic Classification and Functional Review of Autotransporters. Front Immunol 13.
3. Suchland RJ, Carrell SJ, Ramsey SA, Hybiske K, Debrine AM, Sanchez J, Celum C, Rockey DD. 2022. Genomic Analysis of MSM Rectal *Chlamydia trachomatis* Isolates Identifies Predicted Tissue-Tropic Lineages Generated by Intraspecies Lateral Gene Transfer-Mediated Evolution. Infect Immun 0:e00265-22.
4. Holm L, Laiho A, Törönen P, Salgado M. 2023. DALI shines a light on remote homologs: One hundred discoveries. Protein Sci 32:e4519
5. wwPDB consortium. 2019. Protein Data Bank: the single global archive for 3D macromolecular structure data. Nucleic Acids Research 47:D520-D528.
